# Unveiling the ESIPT Luminescence Mechanism of 4′-N,N-Diethylamino-3-Hydroxyflavone in Ionic Liquid: A Computational Study

**DOI:** 10.3390/molecules30061381

**Published:** 2025-03-20

**Authors:** Jin Yang, Qi Li, Meilin Guo, Lu Yan, Lixia Zhu, Jing Zhao, Guangxiong Hu, Hang Yin, Ying Shi

**Affiliations:** Institute of Atomic and Molecular Physics, Jilin University, Changchun 130012, China; yangjin22@mails.jlu.edu.cn (J.Y.); liqi20@mails.jlu.edu.cn (Q.L.); guoml24@mail.jlu.edu.cn (M.G.); yan_lu21@mails.jlu.edu.cn (L.Y.); lixia21@mails.jlu.edu.cn (L.Z.); zhaojing22@mails.jlu.edu.cn (J.Z.); hugx22@mails.jlu.edu.cn (G.H.)

**Keywords:** ESIPT, ionic liquid, solvation effect, dual phosphorescence, DFT/TDDFT

## Abstract

Excited state intramolecular proton transfer (ESIPT) within molecules in solvents plays important roles in photo-chemistry and photo-biology. Herein, the influence of 1-ethyl-3-methyl-imidazolium bis (trifluoromethylsulfonyl) imide ([EMIm][NTf_2_]) and 1-butyl-3-methylimidazolium hexafluorophosphate ([BMIm][PF_6_]) on the ESIPT of 4′-N,N-diethylamino-3-hydroxyflavone (DEAHF) was explored. The density functional theory and time-dependent density functional theory methodologies were used. The calculated fluorescence spectrum reveals that the fluorescence peaks of DEAHF in [EMIm][NTf_2_] and [BMIm][PF_6_] originate from the emission of N* and T* forms. The structure’s optimization, infrared spectra, non-covalent interactions and the scanning of potential energy curves collectively demonstrate that the ESIPT of DEAHF likely happen more in [EMIm][NTf_2_] than in [BMIm][PF_6_]. The solvation effects in [BMIm][PF_6_] exhibit greater prominence compared to those in [EMIm][NTf_2_], as evidenced by the free energy curve. The alterations in dipole moment indicate a substantial solvation relaxation during the ESIPT processes. Our aforementioned research offers backing for the advancement of novel fluorescent probes.

## 1. Introduction

Excited state intramolecular proton transfer (ESIPT) has great importance in the photo-chemistry and photo-biology disciplines [1]. Numerous theoretical and experimental findings demonstrate that the solvents have a clear impact on the properties of ESIPT molecules [2]. It has been reported that the ESIPT dynamics in ionic liquids exhibit a distinct behavior compared to those in traditional solvents, owing to their unique solvation properties and designability [3,4,5]. Specifically, researchers regulate the ESIPT process of molecules through modifying the characteristics of ionic liquids, which has been widely studied [6,7,8]. For instance, the ESIPT process of 1,8-dihydroxyanthraquinone dye in protic ionic liquid solvents can be influenced by solvation effects, which is investigated through the application of steady-state and time-resolved spectroscopic methodologies [9]. Dey et al. modified the physical characteristics of curcumin within micellar aggregates through the introduction of ionic liquids and a commonly used anionic surfactant (SDS) [10]. The investigation revealed that the dynamics of ESIPT processes can be regulated by the alkyl chain length of the ionic liquids and SDS. In Villanueva et al.’s work, they demonstrated that the ESIPT phenomenon of 4′-N, Ndialkylamino-3-hydroxyflavone with varying alkyl chain lengths in ionic liquids is associated with the excitation wavelength [11]. Hessz et al. systematically investigated the ESIPT process of 4′-diethylamino-3-hydroxyflavone (FET) molecules in water and water–acetone mixtures by quantum chemical calculations. The investigation revealed that the intramolecular hydrogen bond in the excited state FET–water complex is weaker, which hinders the ESIPT process [12].

Over the past few years, 4′-N, N-diethylamino-3-hydroxyflavone (DEAHF) has emerged as a prototype molecule with ESIPT, garnering widespread attention [13,14]. The equilibrium S_0_ state of DEAHF only exists as the N form in solutions, which absorbs in the near-UV region [15,16]. Furthermore, DEAHF displays double fluorescence in the majority of solvents due to a swift ESIPT reaction. This process results in N* fluorescence emission at approximately 500 nm and T* form fluorescence emission at around 570 nm [17]. Next, the mechanisms of the dual fluorescence phenomenon of DEAHF in propylene carbonate, acetonitrile and the combinations of propylene carbonate and acetonitrile were theoretically explored by Chen et al. [18]. The research found that modifying the solvent combination is the key to controlling molecular luminescence. Ghosh et al. investigated the ESIPT dynamics of 4′-N,N-Dimethylamino-3-hydroxyflavone in hydrogen-bonding solvents and aqueous micelles by steady-state and time-resolved spectroscopies [19]. The studies revealed that the ESIPT here also correlates well with the solvation dynamics in the hydrophilic shell. In recent years, using an optical Kerr gate technique, the solvation dynamics of DEAHF in ionic liquids were measured [20]. The ESIPT process of DEAHF is affected by ionic liquids 1-ethyl-3-methyl-imidazolium bis(trifluoromethylsulfonyl)imide ([EMIm][NTf_2_]) and 1-butyl-3-methylimidazolium hexafluorophosphate ([BMIm][PF_6_]) with different solvation effects. The solvation effect can influence the ESIPT process of molecules. However, the effect of the solvation of [EMIm][NTf_2_] and [BMIm][PF_6_] on the ESIPT of DEAHF molecules is not clear. Therefore, it is essential to conduct a theoretical investigation of the ESIPT process of DEAHF in [EMIm][NTf_2_] and [BMIm][PF_6_].

**Figure 1:**
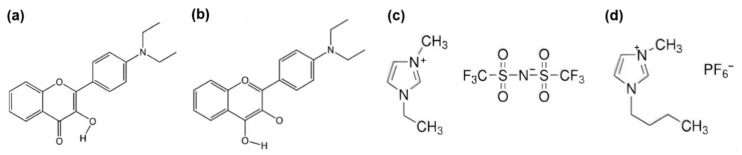
Molecular structures of (**a**) DEAHF of N forms, (**b**) DEAHF of T forms, (**c**) [EMIm][NTf_2_] and (**d**) [BMIm][PF_6_].

In this study, we employ the density functional theory (DFT) and time-dependent density functional theory (TDDFT) to expound the influence of solvation on the ESIPT phenomenon for DEAHF within [EMIm][NTf_2_] and [BMIm][PF_6_] (the structure is shown in Figure 1). Additionally, the analysis of the optimized geometric structures, infrared spectrum, absorption and fluorescence spectra, non-covalent interactions, free energy, dipole moment and potential energy curve is conducted. By analyzing the determined bond lengths, bond angles, non-covalent interactions and infrared spectrum the relative strengths of excited-state hydrogen bonds are compared. Furthermore, the potential for the occurrence of ESIPT is determined by analyzing the potential energy curves. The size of the solvent effect is compared by analyzing free energy and dipole moments. This study seeks to deepen our understanding of the ESIPT process and the influence of solvation on ESIPT for the DEAHF molecule in two distinct ionic liquids.

## 2. Results and Discussion

### 2.1. Optimization of DEAHF Structure

First, the molecular structure of DEAHF was optimized in [EMIm][NTf_2_] and [BMIm][PF_6_]. Analyzing the vibration frequency, the absence of an imaginary frequency in the structure confirmed that the optimized configuration represented a local minimum.

The intramolecular hydrogen bond and bond angle parameters of DEAHF are detailed in Table 1. It is noteworthy that, for the N form the O_2_-H_1_ bond lengths of DEAHF in the S_0_ state were 1.98 Å and 2.00 Å in [EMIm][NTf_2_] and [BMIm][PF_6_], respectively. Meanwhile, in the S_1_ state the O_2_-H_1_ bond lengths reduced to 1.80 Å and 1.84 Å, respectively. Similarly, the bond lengths of O_1_-H_1_ in the S_0_ state were both 0.98 Å in two different ionic liquids. The bond lengths of O_1_-H_1_ were increased to 1.00 Å and 0.99 Å in the S_1_ state, respectively. Based on the aforementioned analysis, it was evident that the hydrogen bond interaction of DEAHF was strengthened in the S_1_ state. It is evident from Table 1 that the bond angles of O_1_-H_1_⋯O_2_ increased from 119.87° and 119.03° in the S_0_ state to 126.37° and 124.68° in the S_1_ state, respectively. It was observed that the bond angle of the S_1_ state approached 180° in contrast to the S_0_ state. This served as further corroboration of the reinforcement of hydrogen bonds in the S_1_ state. In conjunction with the aforementioned alterations in bond length and bond angle, it was noted that the O_2_-H_1_ bond length in [EMIm][NTf_2_] was shorter and the bond angle was closer to 180° than that in [BMIm][PF_6_]. This indicated that the hydrogen bonds were stronger and that ESIPT was more likely to occur in [EMIm][NTf_2_]. For the T form, the ESIPT processes caused the formation of a hydrogen bond between H_1_ and O_2_, resulting in bonding with O_1_. The bond length of O_2_-H_1_ was decreased while that of O_1_-H_1_ was increased in the S_1_ state, with changes of 0.02 Å and 0.12 Å, respectively, in [EMIm][NTf_2_]. The O_2_-H_1_ bond length of DEAHF in [BMIm][PF_6_] decreased by 0.01 Å, whereas the O_1_-H_1_ bond length increased by 0.11 Å. Through the above analysis of bond lengths, it was found that the structure of the DEAHF molecule became more stable in [BMIm][PF_6_] than in [EMIm][NTf_2_] after the ESIPT process.

### 2.2. Absorption and Fluorescence Spectra

We used the TZVP basis set and B3PW91 functional to calculate the absorption fluorescence spectrum of DEAHF. Found in Table 2, the absorption peak values were 426 nm and 427 nm in [EMIm][NTf_2_] and [BMIm][PF_6_], which were consistent with the respective experimental values of ~411 nm and ~411 nm [20]. The fluorescence spectrum of the DEAHF molecule was calculated and analyzed, revealing two distinct fluorescence peaks corresponding to the N* and T* forms of DEAHF. Next, the fluorescence peak values in the short wave region were 488 nm and 490 nm, while the experimental values were ~530 nm and ~524 nm [20]. After going through ESIPT, the fluorescence emission spectrum values were 612 nm and 608 nm, respectively. The experimental values were ~589 nm and ~578 nm [20]. Compared with other functionals we have used, the currently employed functional, B3PW91, provides the best match. Therefore, this method is used to calculate DEAHF.

### 2.3. Infrared (IR) Spectra Analysis

**Figure 2:**
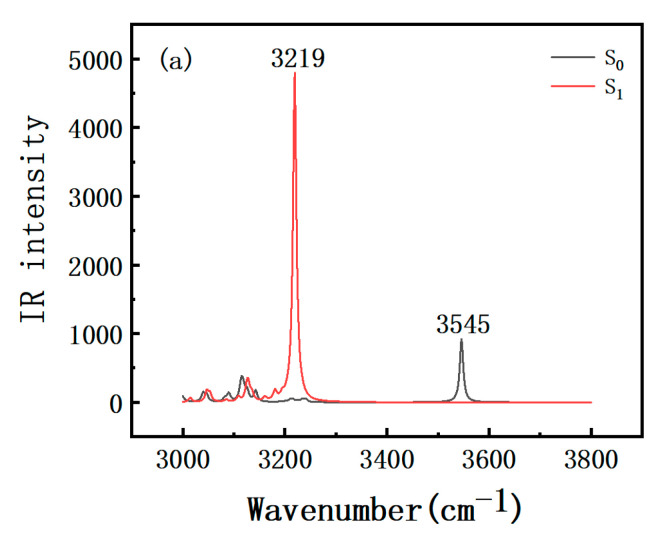

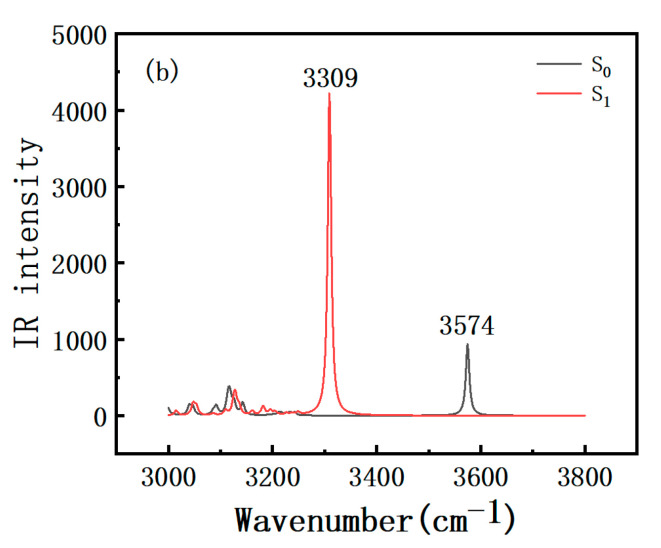
Calculated IR spectra of DEAHF molecule in its N form in S_0_ and S_1_ states in [EMIm][NTf_2_] (**a**) and [BMIm][PF_6_] (**b**). Black line represents S_0_ state, and red line represents S_1_ state.

For the purpose of reflecting the relevant information on hydrogen bond dynamics of the DEAHF in [EMIm][NTf_2_] and [BMIm][PF_6_], the infrared spectra for both the S_0_ and S_1_ states within the vibration regions of the O_1_-H_1_ stretching group were calculated theoretically [22,23]. Figure 2 illustrates the vibration frequencies in the S_0_ and S_1_ states. In the S_0_ state, the O_1_-H_1_ vibration peak of DEAHF was observed at 3545 cm^−1^ in [EMIm][NTf_2_] and at 3574 cm^−1^ in [BMIm][PF_6_]. Upon photo-excitation, the stretching vibration frequencies of DEAHF in the S_1_ state were 3219 cm^−1^ and 3309 cm^−1^ in [EMIm][NTf_2_] and [BMIm][PF_6_], respectively. Therefore, the IR vibration spectrum of DEAHF exhibited a red shift from the S_0_ state to the S_1_ state when dissolved in two distinct ionic liquids. The detected increase in the red shift of the stretching vibration peak can be utilized as a marker to indicate the strengthening of the hydrogen bond in its excited state [24]. It is worth mentioning that the peak position of the infrared vibration spectrum of O_1_-H_1_ in [BMIm][PF_6_] (3309 cm^−1^) shifts to a blue position relative to that of [EMIm][NTf_2_] (3219 cm^−1^) in the S_1_ state. That is to say, the hydrogen bond interaction was stronger in [EMIm][NTf_2_] than [BMIm][PF_6_]. And the ESIPT process of DEAHF was observed to be more probable to take place in [EMIm][NTf_2_] than in [BMIm][PF_6_].

### 2.4. Non-Covalent Interaction (NCI) Analysis

Yang et al. proposed non-covalent interactions as a straightforward visual method to explore hydrogen bond characteristics [25,26]. By analyzing the electron density (*ρ*(r)) and density gradient reduction (RDG) profiles, various categories of interactions and their strengths can be visualized in the physical realm. The Equation (1) representing the RDG function can be formulated as

(1) $$RDG(r)=\frac{1}{2{\left(3\pi^{2}\right)}^{1/3}}\frac{\left|\nabla \rho (r)\right|}{\rho {\left(r\right)}^{4/3}}$$

In addition, based on the Bader atoms in molecules theory, the electron density matrix and the second largest eigenvalue $\lambda_{2}$ for ρ(r) can be accessed in Equation (2)

(2) $$\Omega (r)=sign(\lambda_{2}(r))\rho (r)$$

**Figure 3:**
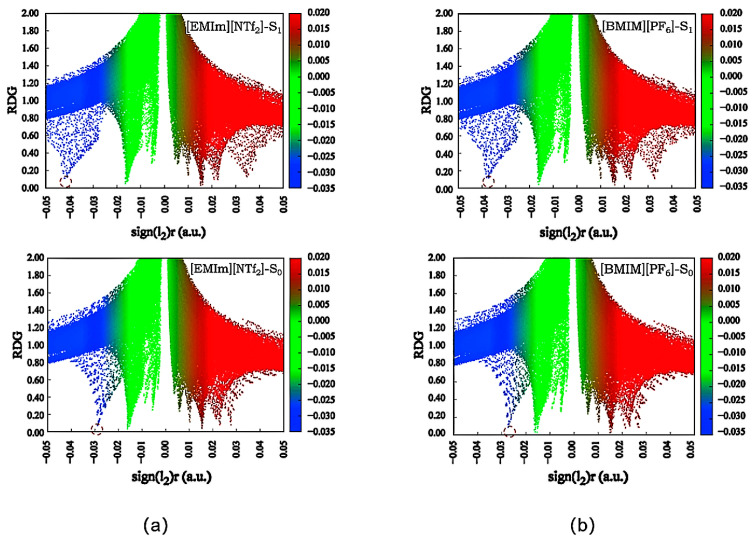
RDG scatter plots of DEAHF molecules in (**a**) [EMIm][NTf_2_] and (**b**) [BMIm][PF_6_].

The parameter λ_2_ > 0 signifies bonding interactions, whereas λ_2_ < 0 denotes anti-bonding interactions. A negative value of Ω(r) indicates a hydrogen bond interaction, while a positive value of Ω(r) corresponds to a steric repulsion interaction. When the value of Ω(r) approaches zero, it signifies van der Waals interaction [27]. Scatter plots of the X(r) versus RDG(r) of DEAHF in [EMIm][NTf_2_] and [BMIm][PF_6_] were executed, as illustrated in Figure 3. In the S_0_ state, the peak value of DEAHF in the two different ionic liquids was between −0.027 and −0.030, and the peak position was almost the same. The findings indicated that the intramolecular interaction of the ground state of DEAHF remained essentially unchanged. In the excited state, the peak position of DEAHF was shifted to −0.043 and −0.037 in [EMIm][NTf_2_] and [BMIm][PF_6_], respectively. The red shift proved that the strength of the O_2_-H_1_ bond interaction in DEAHF was increased in the S_1_ state. It was obvious that the O_2_-H_1_ bond interaction in [EMIm][NTf_2_] was stronger than that in [BMIm][PF_6_]. Therefore, the ESIPT process was more probable to take place in [EMIm][NTf_2_]. In addition, we also calculated the hydrogen bond strength using the QTAIM theory by Multiwfn [28,29,30,31]. In the ionic liquid [EMIm][NTf_2_], the value of electron density ρ(r) in the ground state was 0.0288 (a.u.), and that in the excited state was 0.0423 (a.u.). The value of Laolacian ∇^2^ρ(r) in the ground state was 0.1145 (a.u.), and that in the excited state was 0.1291 (a.u.). The bond energy of the hydrogen bond was also calculated by using the equation−223.08 × ρ/au + 0.7423 kcal/mol [32]. The strength of the hydrogen bond in the ground state was −5.7 kcal/mol, and that in the excited state was −8.7 kcal/mol. In the ionic liquid [BMIm][PF_6_], the value of electron density ρ(r) in the ground state was 0.0275 (a.u.), and that in the excited state was 0.0382 (a.u.). The value of Laolacian ∇^2^ρ(r) in the ground state was 0.1131 (a.u.), and that in the excited state was 0.1258 (a.u.). The strength of the hydrogen bond in the ground state was −5.4 kcal/mol, and that in the excited state was −7.8 kcal/mol. When the value gets larger, the hydrogen-bond strength gets stronger. The results indicate that the hydrogen bond interaction was stronger in [EMIm][NTf_2_] than [BMIm][PF_6_]. And the ESIPT process of DEAHF occurs more readily in [EMIm][NTf_2_] than in [BMIm][PF_6_].

### 2.5. Potential Energy Curves

**Figure 4:**
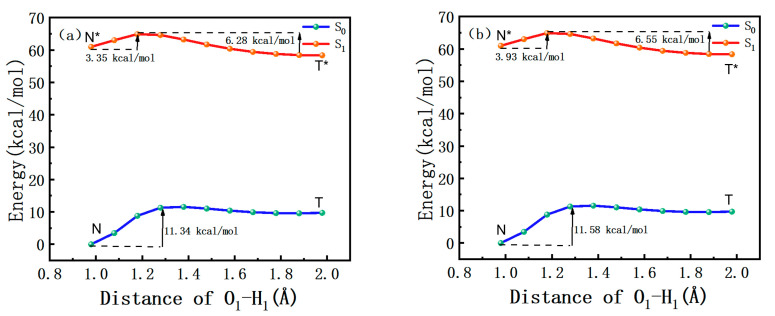
Potential energy curves of DEAHF in (**a**) [EMIm][NTf_2_] and (**b**) [BMIm][PF_6_] along with O_1_-H_1_ bond length in S_0_ and S_1_ states.

In order to investigate whether ESIPT could occur in [EMIm][NTf_2_] and [BMIm][PF_6_], systematic studies were conducted on the ESIPT potential energy curves of the S_0_ and S_1_ states. The calculations were carried out based on the optimized geometry of their respective electronic states, while maintaining fixed O_1_-H_1_ spacing at various values. The ESIPT potential energy curves for the O_1_-H_1_ bond length ranging from 1.0 to 2.0 Å in increments of 0.1 Å are depicted in Figure 4. The energy barriers were 11.3 kcal/mol and 11.6 kcal/mol in the ground state in [EMIm][NTf_2_] and [BMIm][PF_6_]. The high energy barriers confirmed the conclusion that the transfer of H_1_ from the DEAHF molecule to O_2_ was hindered by a nearly insurmountable barrier in the S_0_ state. Conversely, the barriers of DEAHF in the S_1_ state were lower than the one in the S_0_ state. The energy barriers of the S_1_ state were 3.4 kcal/mol and 3.9 kcal/mol in [EMIm][NTf_2_] and [BMIm][PF_6_]. A reduced energy barrier suggested that the ESIPT process of DEAHF was more probable to occur in the first excited state as opposed to the ground state. Concurrently, the backward barriers were 6.3 kcal/mol ([EMIm][NTf_2_]) and 6.6 kcal/mol ([BMIm][PF_6_]), respectively. The elevated energy barriers in the reverse direction indicated that the backward ESIPT process was likely to be hindered. Consequently, the conversion of H_1_ to O_2_ for the DEAHF molecule was expected to occur predominantly in the S_1_ state. Through a comparison of the barriers of DEAHF in the S_1_ state, it was discerned that the energy barriers in [EMIm][NTf_2_] exhibited lower values compared to those in [BMIm][PF_6_]. It could also be observed that the DEAHF in [EMIm][NTf_2_] was more prone to the ESIPT process than in [BMIm][PF_6_].

### 2.6. Free Energy

**Figure 5:**
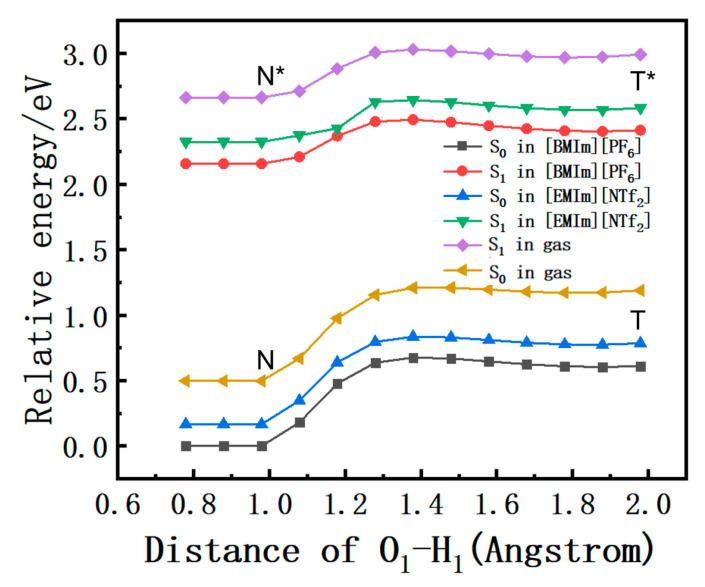
Relative energy as a function of the distance of O_1_-H_1_. The different lines represent the free energy in gas. [EMIm][NTf_2_] and [BMIm][PF_6_] correspond to the S_0_ and S_1_ states, respectively.

The exceptional solvation effect of ionic liquids arises from the robust interaction between ionic liquids and solute molecules, as well as the presence of bulky cations and highly polarized anions within the ionic liquids [33]. So, the free energy curve was calculated for DEAHF in different ionic liquids. And the energy distribution of the ground state and first excited states in the gas phase for [EMIm][NTf_2_] and [BMIm][PF_6_] along the O_1_-H_1_ distance is illustrated in Figure 5. In the first excited state, dual minima were identified in two different ionic liquids, which corresponded to the experimental observation of N* and T* fluorescence spectra [20]. From Figure 5, it is evident that the relative energy of the ground state and first excited state in the different phases increased with the elongation of O_1_-H_1_, while maintaining a similar free energy curve profile. Moreover, the relative energy of the N configuration was found to be markedly decreased than that of the T configuration, implying a higher stability of the DEAHF molecule in its N form. Additionally, the relative energy of the first excited state was higher than the ground state in both of the two different ionic liquids. The discrepancy between the gas and liquid phases’ curves, depicted in Figure 5, corresponds to the solvation effect in energy. The relative energy was observed to be lower in the liquid phase, with [BMIm][PF_6_] demonstrating notably diminished values compared to [EMIm][NTf_2_]. These results implied that the DEAHF molecule had a considerable solvation effect in [BMIm][PF_6_] compared to [EMIm][NTf_2_].

### 2.7. Dipole Moment

**Figure 6:**
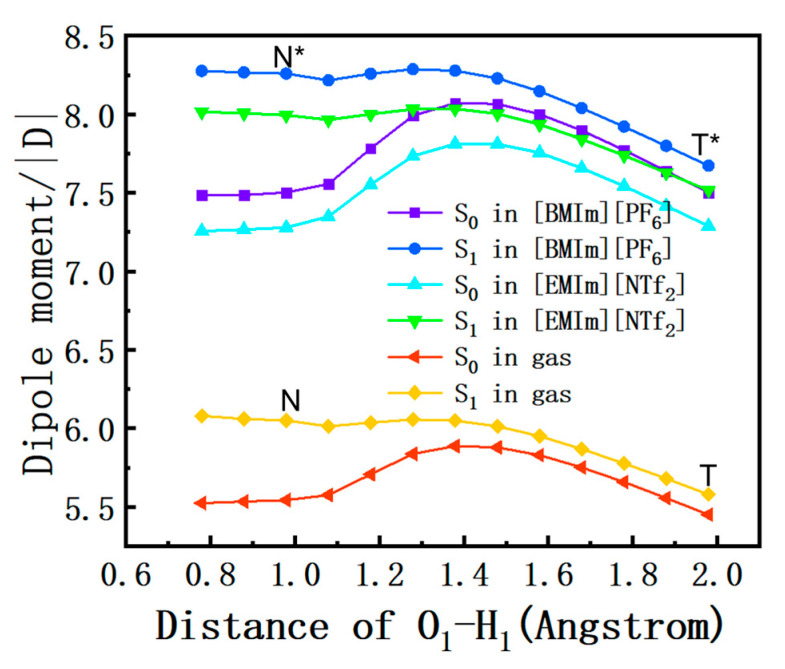
The changes in dipole moment along the distance of O_1_-H_1_. The different lines represent the dipole moment in gas, and [EMIm][NTf_2_] and [BMIm][PF_6_] correspond to the S_0_ state and the S_1_ state, respectively.

The solvation effect on the free energy distribution could be explained by the dipole moment of the DEAHF. The calculation of the dipole moment along the O_1_-H_1_ distance is depicted in Figure 6. It was evident that the dipole moment increased from S_0_ to S_1_ in the N form, resulting in an intensified electrostatic interaction between the solute and solvent [34]. In the S_1_ state, the dipole moment in the liquid phase exhibited a significantly greater magnitude compared to that in the gas phase. Specifically, the dipole moment of [BMIm][PF_6_] exceeded that of [EMIm][NTf_2_]. However, as the ESIPT reaction progresses, the dipole moment of the excited state rapidly decreased, making the dipole moment of the T form comparable to that of the S_0_ state. The increase in the dipole moment of the N form of the DEAHF molecule induced by excitation could be understood by the perspective of the transfer of electrons from the aniline moiety to the chromene moiety [18]. This results in an approximate increase of 0.7 D in the dipole moment of the N form of the DEAHF molecule upon excitation. Additionally, it was observed that the electronegativity of the O_2_ atom exceeded that of the O_1_ atom. The change in the charge distribution provided the driving force for the ESIPT process in the DEAHF molecule. The results showed once again that DEAHF molecule had a greater solvation effect in [BMIm][PF_6_] than in [EMIm][NTf_2_]. In addition, the dipole moment can be estimated using the following equation in experiment [35]:

(3) $$\Delta_{\mu }=\sqrt{\frac{mX81}{{\left(6.2/a\right)}^{3}11307.6}}$$

The parameter m represents the slope of the Stokes shift, $\Delta_{\mu }$ represents the change in dipole moment from the ground state to the excited state, a is the Onsager cavity radius and X represents constant. Therefore, the Stokes shift can be derived from the variation in the dipole moment. Since the peak positions of the calculated absorption peaks are nearly the same in both ionic liquids, an increase in the dipole moment change corresponds to the redshift of the fluorescence peaks. As shown in Table 3, the changes in the dipole moment variation for the same structure in both ionic liquids were calculated to be 0.05 and 0.05 D. Although there is a more pronounced difference in the calculated dipole moments in the two ionic liquids, the small Δ values of dipole moment variation lead to insignificant changes in the luminescent characteristic.

## 3. Computational Methods and Details

The Gaussian 16 [36] software was utilized for performing DFT/TD-DFT calculations. We employed the TZVP [37] basis set along with various functionals (CAM-B3LYP [38], PBEPBE [21], B1B95 [39], WB97XD [40], B3PW91 [41] and mPW1PW91 [42]) to optimize the structure of the DEAHF molecule by SMD method in [EMIm][NTf_2_] and [BMIm][PF_6_]. By comparing the absorption peaks and fluorescence peaks with the experimental value, the functional of B3PW91 with TZVP basis set was determined. All calculations were performed with a (75, 302) pruned grid. The SCF convergence criterion on root mean square density matrix differences was fixed to 10^−8^. In addition, the impact of the ionic liquids’ solvent properties was considered in the SCRF calculation by employing the SMD model [43]. The SMD model has been shown to provide a reliable description of solvent effects in ionic liquid solutions [44]. Regarding the solvent descriptors used in SMD-calculations, we have put them in Table S1. The S_0_ state geometry of the DEAHF was optimized without imposing any restrictions on bonds or angles. The optimized DEAHF molecule was used as the initial configurations to achieve the vertical excitation energy. The next calculation was going to be based on the geometry that was optimized before. The geometry of the S_1_ state was optimized based on the structure of the optimized S_0_ state. The absence of imaginary frequencies ascertained the nature of these points as minimal. Fluorescence emission energies were calculated based on the excited state configurations, i.e., fluorescence spectra were acquired [45]. Furthermore, the potential energy curves were obtained by keeping the O_1_-H_1_ distance fixed and the step size increasing. The non-covalent interactions were calculated using the Multiwfn 3.8 program by RDG function [28].

## 4. Conclusions

In summary, the present work focused on performing a comprehensive theoretical analysis of the ESIPT process for the DEAHF molecule in two different ionic liquids. The determined bond distances, bond angles, non-covalent interactions and infrared vibration spectra indicated that the hydrogen bond strength in [EMIm][NTf_2_] was greater than that in [BMIm][PF_6_] in the first excited state. The stronger the hydrogen bond strength, the more likely the ESIPT process will occur. Moreover, by calculating the ESIPT potential energy curves, it was observed that in the S_1_ state, the energy barrier of DEAHF in [EMIm][NTf_2_] was 3.35 kcal/mol, which is lower than that of 3.93 kcal/mol in [BMIm][PF_6_]. This shows that the DEAHF was more prone to undergoing ESIPT in [EMIm][NTf_2_] compared to [BMIm][PF_6_]. Subsequently, analyzing the free energy curves revealed that the relative energy of [BMIm][PF_6_] was slightly lower than that of [EMIm][NTf_2_]. It can be concluded that the solvation effect of [BMIm][PF_6_] was larger than that of [EMIm][NTf_2_]. This was further demonstrated by the subsequent analysis of the dipole moment. Our theoretical calculations deepen the understanding of how ionic liquids affect the microscopic ESIPT mechanism. This lays a foundation for regulating molecular luminescence when using ionic liquids as solvents in the future.

## Figures and Tables

**Table 1:** Bond lengths (Å) and angles (deg) of N and T forms in S_0_ and S_1_, respectively, in [EMIm][NTf_2_] and [BMIm][PF_6_] by B3PW91 calculations.

		N		T	
		S_0_	S_1_	S_0_	S_1_
DEAHF in [EMIm][NTf_2_]	O_1_-H_1_	0.98	1.00	1.81	1.95
	O_2_-H_1_	1.98	1.80	1.00	0.98
	δ(O_1_-H_1_⋯O_2_)	119.87	126.37	125.03	120.16
DEAHF in [BMIm][PF_6_]	O_1_-H_1_	0.98	0.99	1.86	1.97
	O_2_-H_1_	2.00	1.84	0.99	0.98
	δ(O_1_-H_1_⋯O_2_)	119.03	124.68	123.14	119.16

**Table 2:** The absorption and fluorescence peaks (nm) of the DEAHF in [EMIm][NTf_2_] and [BMIm][PF_6_]. (Exp: experimental spectral value).

		Structures	mPW1PW91	CAM-B3LYP	WB97XD	PBEPBE	B1B95	B3PW91	Exp. ^a^
[EMIm][NTf_2_]	absorption	N	407	364	342	400	400	426	~411
		T	501	457	449	513	503	522	-
	fluorescence	N	460	443	476	469	464	488	~530
		T	604	591	593	600	596	612	~589
[BMIm][PF_6_]	absorption	N	407	353	341	408	402	427	~411
		T	502	453	449	519	502	519	-
	fluorescence	N	470	450	481	471	466	490	~524
		T	602	589	589	596	591	608	~578

^a^ Literature [21].

**Table 3:** The changes in the dipole moment variation (D) of the DEAHF in [EMIm][NTf_2_] and [BMIm][PF_6_].

[EMIM][NTf_2_]	N	T
S_0_	7.28	7.29
S_1_	7.99	7.51
△_1_	0.71	0.22
[BMIM][PF_6_]	N	T
S_0_	7.50	7.50
S_1_	8.26	7.67
△_2_	0.76	0.17
△_1_ − △_2_	0.05	0.05

△_1,_ △_2_: the difference between the dipole moment of the S_1_ state and that of the S_0_ state.

## Data Availability

Data are contained within the article and Supplementary Materials.

## Supplementary Materials

The following supporting information can be downloaded at: https://www.mdpi.com/article/10.3390/molecules30061381/s1, Table S1: The parameters of ionic liquids [EMIm][NTf2] and [BMIm][PF6].
