# Supplementary material for: Unveiling the ESIPT Luminescence Mechanism of 4′-N,N-Diethylamino-3-Hydroxyflavone in Ionic Liquid: A Computational Study
Source: Molecules. 2025 Mar 20;30(6):1381. doi: 10.3390/molecules30061381 (PMC11945155; doi:10.3390/molecules30061381)
Supplement: Supplementary file 1 [file molecules-30-01381-s001.zip › molecules-3444181-supplementary.pdf]

1. We employed the TZVP basis set along with B3PW91 functionals （with SMD）：

Computed SI coordinates of structure of DEAHF in [EMIm][NTf$_2$] in N form (S$_0$ state)

C	-3.74777900	-2.26755000	0.04136200
C	-3.21515000	-0.97893600	0.01055600
C	-4.04104200	0.14808200	0.02714700
C	-5.43127600	-0.03468400	0.07204800
C	-5.96597100	-1.30427200	0.10212100
C	-5.11880200	-2.42081000	0.08650900
C	-3.42070200	1.45924300	-0.01045300
C	-1.97855400	1.47565000	-0.03642400
C	-1.23012300	0.32943800	-0.06414800
O	-1.86673100	-0.87624800	-0.04052600
C	0.21464800	0.19624800	-0.09474200
C	0.82236400	-1.04283600	0.16459600
C	2.19099800	-1.20675900	0.13666600
C	3.06056700	-0.13395800	-0.17605800
C	2.44289200	1.11735700	-0.41276300
C	1.07390600	1.27162800	-0.37954300
N	4.41801800	-0.30355700	-0.25780100
C	5.29953200	0.85811800	-0.29345800
C	5.53181700	1.50602300	1.06631300
C	4.98732400	-1.60553200	0.10279000
C	6.44222500	-1.79909900	-0.28247500
O	-4.03201100	2.54149800	-0.00093900
O	-1.43125800	2.71318100	-0.03625500
H	-2.21023200	3.30730000	-0.01512300
H	-3.08063200	-3.12131700	0.02814200
H	-6.06676900	0.84319000	0.08430500

H	-7.04032500	-1.44286700	0.13740100
H	-5.54139000	-3.41902500	0.10996600
H	0.20850600	-1.90086200	0.40832200
H	2.58648700	-2.18446400	0.37155600
H	3.04079600	1.99035700	-0.63367000
H	0.66299600	2.25111700	-0.57886200
H	6.25055200	0.54191300	-0.71798300
H	4.89629200	1.58616700	-0.99826100
H	6.20196700	2.36399800	0.96271900
H	5.99185700	0.80254500	1.76497700
H	4.59659900	1.85943900	1.50700100
H	4.86806800	-1.78656900	1.18027500
H	4.40582100	-2.37384500	-0.41093400
H	6.71539300	-2.83405200	-0.06242400
H	7.12275800	-1.15652900	0.27917300
H	6.60388300	-1.63315000	-1.35048600

Computed SI coordinates of structure of DEAHF in [EMIm][NTf_2] in T form (S_0 state)

C	-3.74234500	-2.27127600	0.18801900
C	-3.20636500	-0.98884700	0.08051100
C	-4.02926600	0.14911300	0.01169800
C	-5.43016400	-0.02984300	0.05688500
C	-5.95717700	-1.29399800	0.16306500
C	-5.11270300	-2.41675600	0.22922900
C	-3.36746600	1.38558600	-0.09261800
C	-1.95068000	1.51409600	-0.13787600
C	-1.21245900	0.30718300	-0.06100100
O	-1.86629600	-0.87477200	0.04251800
C	0.21643000	0.17329200	-0.08702900

C	0.83841900	-1.08901700	-0.01433700
C	2.20616100	-1.22934600	-0.04203700
C	3.06729000	-0.10732800	-0.16267400
C	2.43742700	1.16136700	-0.20306100
C	1.06805100	1.29370400	-0.17621300
N	4.42395600	-0.24912600	-0.24490300
C	5.29461500	0.91426900	-0.11228000
C	5.52281000	1.35633200	1.32795600
C	5.00767900	-1.58737200	-0.10033800
C	6.46830200	-1.69927000	-0.49489700
O	-4.00915400	2.53979000	-0.16379600
O	-1.49281300	2.70387000	-0.23914900
H	-3.24173500	3.17775900	-0.23082200
H	-3.07636000	-3.12452200	0.23796900
H	-6.07374500	0.84004100	0.00587300
H	-7.03193600	-1.43037200	0.19694200
H	-5.54167200	-3.40860600	0.31325000
H	0.23286600	-1.98211500	0.07704500
H	2.61336800	-2.22718200	0.03663900
H	3.02908400	2.06433300	-0.25893900
H	0.62259000	2.27871000	-0.21917000
H	6.24754000	0.67304200	-0.57933400
H	4.88089000	1.73283000	-0.70188400
H	6.18127500	2.22911600	1.35038300
H	5.99469400	0.56356200	1.91387300
H	4.58454600	1.62826400	1.81710900
H	4.88005800	-1.94300000	0.93120300
H	4.44036600	-2.26593800	-0.74030200
H	6.75220400	-2.75298000	-0.43810400
H	7.13481700	-1.14634100	0.16935200

H	6.63821200	-1.36516300	-1.52135300

Computed SI coordinates of structure of DEAHF in [EMIm][NTf_2] in N form (S_1 state)

C	-3.75167800	-2.25525700	0.19653500
C	-3.21997700	-0.98433700	0.08313100
C	-4.05061700	0.16082700	0.00981800
C	-5.45287500	-0.04000100	0.05669800
C	-5.98148600	-1.31392000	0.16905700
C	-5.14169000	-2.42509700	0.23953300
C	-3.44466300	1.43950400	-0.10042900
C	-1.98402800	1.46089100	-0.13679700
C	-1.20824100	0.32622700	-0.05678400
O	-1.85604100	-0.89448800	0.04509000
C	0.22019900	0.20376300	-0.08137200
C	0.83200900	-1.07535800	-0.04036000
C	2.19556100	-1.22901400	-0.07322600
C	3.06524100	-0.10841600	-0.15207000
C	2.45107700	1.17573600	-0.16779400
C	1.08911000	1.32286000	-0.13503800
N	4.41856100	-0.25805000	-0.21069000
C	5.30701400	0.89523800	-0.12274200
C	5.58109700	1.33134000	1.31330000
C	4.99380400	-1.60805600	-0.14379500
C	6.46923300	-1.71084100	-0.47128200
O	-4.02048900	2.56871800	-0.17632000
O	-1.50096100	2.69921200	-0.24881300
H	-2.34786700	3.22790000	-0.27052100
H	-3.08103900	-3.10521200	0.25017000
H	-6.09652300	0.82990900	0.00169200

H	-7.05758900	-1.44864800	0.20268800
H	-5.55600900	-3.42278600	0.32777200
H	0.20992200	-1.95774200	0.01997100
H	2.59536200	-2.23180400	-0.02754100
H	3.05439000	2.07146500	-0.20157500
H	0.67723400	2.32141300	-0.15145400
H	6.24014400	0.63324000	-0.61572400
H	4.88055500	1.71576100	-0.69815800
H	6.25777200	2.18954400	1.30670400
H	6.05259900	0.53009300	1.88623500
H	4.66016600	1.62404000	1.82182100
H	4.80992800	-2.01603900	0.85847200
H	4.44178000	-2.23522100	-0.84689900
H	6.73922700	-2.76873100	-0.43395900
H	7.10351700	-1.18595600	0.24454700
H	6.69189000	-1.34893200	-1.47737200

Computed SI coordinates of structure of DEAHF in [EMIm][NTf_2] in T form (S_1 state)

C	-3.73474100	-2.26532800	0.18360400
C	-3.20192900	-0.99318500	0.07831900
C	-4.03881300	0.15116200	0.01104700
C	-5.44386500	-0.04650300	0.05419100
C	-5.96614500	-1.32049800	0.15883500
C	-5.12040600	-2.43062400	0.22396200
C	-3.40534600	1.38931300	-0.09065200
C	-1.96805100	1.51630900	-0.13177500
C	-1.19195500	0.32543800	-0.05953100
O	-1.84997700	-0.88870400	0.04097200
C	0.22029000	0.19934000	-0.08128900

C	0.84434400	-1.07563300	-0.00738200
C	2.20954900	-1.22280000	-0.03782200
C	3.07782000	-0.10513900	-0.15403200
C	2.45474100	1.17346700	-0.19792000
C	1.09205100	1.31833300	-0.16806800
N	4.43338800	-0.25167800	-0.22725600
C	5.31406200	0.90657500	-0.13293000
C	5.56882400	1.36503300	1.29907400
C	5.01091300	-1.59510700	-0.11095400
C	6.47778300	-1.70820800	-0.47723000
O	-4.08953700	2.53379900	-0.16040800
O	-1.51029500	2.69682500	-0.22950400
H	-3.38519500	3.21890900	-0.22770000
H	-3.06535200	-3.11620500	0.23290400
H	-6.09447900	0.81811300	0.00358500
H	-7.04110300	-1.46000200	0.19065000
H	-5.53551800	-3.42862100	0.30620600
H	0.23069500	-1.96229300	0.08224200
H	2.61231200	-2.22302700	0.03783800
H	3.05468300	2.07091400	-0.25597200
H	0.66267100	2.30964000	-0.21024900
H	6.25654100	0.64869600	-0.61131700
H	4.89394500	1.71973500	-0.72485900
H	6.23750800	2.23005800	1.29465000
H	6.04071700	0.57514700	1.88817300
H	4.64016800	1.65480000	1.79571700
H	4.85931900	-1.96830200	0.91121100
H	4.44683400	-2.25519700	-0.77346300
H	6.75333600	-2.76404300	-0.42118000
H	7.13237800	-1.16359600	0.20519700

H	6.67053400	-1.37001200	-1.49799800

Computed SI coordinates of structure of DEAHF in [BMIm][PF_6] in N form (S_0 state)

C	-3.73585800	-2.27069100	0.06926000
C	-3.20949400	-0.98026100	0.02407100
C	-4.03932400	0.14349000	0.02735400
C	-5.42890100	-0.04459400	0.07358500
C	-5.95781600	-1.31602700	0.11796400
C	-5.10609600	-2.42918000	0.11550600
C	-3.42415800	1.45584000	-0.02661800
C	-1.98389800	1.47947000	-0.05610300
C	-1.22969900	0.33551400	-0.06877500
O	-1.86155000	-0.87163400	-0.02848800
C	0.21492900	0.20547200	-0.09899100
C	0.82211400	-1.03896500	0.13640600
C	2.19043600	-1.20291700	0.10750000
C	3.06100900	-0.12430800	-0.18021500
C	2.44462500	1.13195800	-0.39044700
C	1.07576500	1.28617400	-0.35894000
N	4.41893100	-0.29286800	-0.26567100
C	5.30082400	0.86946700	-0.24375600
C	5.51686000	1.46025900	1.14395400
C	4.98621600	-1.60380000	0.06381300
C	6.43782000	-1.79221000	-0.33561700
O	-4.04482000	2.53519700	-0.02996800
O	-1.43656700	2.71819500	-0.07430900
H	-2.20924600	3.31883200	-0.05827400
H	-3.06405900	-3.12084300	0.06518500
H	-6.06917300	0.82988100	0.07483300
H	-7.03156900	-1.45874300	0.15392600

H	-5.52465300	-3.42874400	0.14983600
H	0.20781300	-1.90159200	0.36172900
H	2.58477200	-2.18560800	0.32257300
H	3.04332600	2.00927800	-0.59115600
H	0.66707300	2.26980700	-0.54029800
H	6.25653800	0.57075400	-0.67054700
H	4.90610600	1.62591000	-0.92279100
H	6.18724800	2.32213300	1.08399000
H	5.96947600	0.72889500	1.81842200
H	4.57610600	1.79458800	1.58782800
H	4.87296700	-1.80912800	1.13762200
H	4.39966300	-2.35840100	-0.46382400
H	6.70803200	-2.83432300	-0.14820700
H	7.12493400	-1.16985100	0.24048100
H	6.59349900	-1.59498500	-1.39921100

Computed SI coordinates of structure of DEAHF in [BMIm][PF_6] in T form (S0 state)

C	-3.72605600	-2.27406200	0.21677900
C	-3.19773400	-0.99013500	0.09412100
C	-4.02547100	0.14229900	0.01275700
C	-5.42491700	-0.04217200	0.06851100
C	-5.94532300	-1.30768600	0.19072800
C	-5.09543600	-2.42555700	0.26564200
C	-3.37118100	1.38228300	-0.10475800
C	-1.95766000	1.51722700	-0.17024300
C	-1.21250200	0.31544000	-0.06997400
O	-1.85836800	-0.86845300	0.05196100
C	0.21681700	0.18580000	-0.09895200
C	0.83752900	-1.07903400	-0.07171700

C	2.20500200	-1.21984300	-0.09725000
C	3.06763300	-0.09503600	-0.16807600
C	2.43929000	1.17482000	-0.16268300
C	1.07007800	1.30757500	-0.13899100
N	4.42479600	-0.23481600	-0.24965200
C	5.29480400	0.91965800	-0.04943000
C	5.50418900	1.29112600	1.41309400
C	5.00638200	-1.57809300	-0.14972200
C	6.46938500	-1.67631800	-0.53835500
O	-4.03435200	2.52591800	-0.18654500
O	-1.49750100	2.70563100	-0.29857100
H	-3.29570800	3.18670800	-0.27402100
H	-3.05458500	-3.12251900	0.27361400
H	-6.07275900	0.82400900	0.01020100
H	-7.01923100	-1.44867800	0.23128100
H	-5.51946300	-3.41841000	0.36187000
H	0.23047500	-1.97425100	-0.01989400
H	2.61035400	-2.22069000	-0.05728700
H	3.03172100	2.07884300	-0.17882600
H	0.62893700	2.29497600	-0.14750700
H	6.25356300	0.69999500	-0.51522900
H	4.89002300	1.76623300	-0.60451900
H	6.16252800	2.16115500	1.48631000
H	5.96830200	0.47065800	1.96620200
H	4.55966700	1.53985200	1.90256700
H	4.87148200	-1.97159400	0.86703200
H	4.44340300	-2.23204600	-0.81834700
H	6.75148500	-2.73190000	-0.52280200
H	7.13226800	-1.15228200	0.15240700
H	6.64677500	-1.30086500	-1.54912700

Computed SI coordinates of structure of DEAHF in [BMIm][PF_6] in N form (S_1 state)

C	-3.74789000	-2.25661800	0.19754600
C	-3.21834200	-0.98456000	0.08680600
C	-4.04913900	0.15880600	0.00998600
C	-5.45051800	-0.04363500	0.05009300
C	-5.97794800	-1.31814600	0.16029400
C	-5.13695500	-2.42797000	0.23483100
C	-3.44488200	1.43951300	-0.09724100
C	-1.98726000	1.46444300	-0.13393200
C	-1.20893600	0.33021600	-0.05211900
O	-1.85417800	-0.89171100	0.05520600
C	0.21957100	0.20829000	-0.07842500
C	0.83046800	-1.07147900	-0.03600900
C	2.19369300	-1.22650700	-0.07121800
C	3.06418200	-0.10710100	-0.15382200
C	2.45143500	1.17743300	-0.17038000
C	1.08973300	1.32624000	-0.13521600
N	4.41739500	-0.25823900	-0.21623600
C	5.30702700	0.89378600	-0.12449100
C	5.57966700	1.32660500	1.31253000
C	4.99153600	-1.60823900	-0.14381100
C	6.46607600	-1.71344900	-0.47354600
O	-4.03318200	2.56511500	-0.17297100
O	-1.49360100	2.70023700	-0.24999200
H	-2.32315900	3.24732700	-0.27422400
H	-3.07572300	-3.10519100	0.25372100
H	-6.09621000	0.82460700	-0.00875200
H	-7.05392400	-1.45422900	0.18894100

H	-5.55048000	-3.42611200	0.32134700
H	0.20832400	-1.95360800	0.02709800
H	2.59240200	-2.22967400	-0.02437400
H	3.05545500	2.07255400	-0.20700300
H	0.67972700	2.32535700	-0.15223000
H	6.24045300	0.63207000	-0.61711400
H	4.88266300	1.71603700	-0.69896500
H	6.25692900	2.18431700	1.30853000
H	6.04992500	0.52395800	1.88450600
H	4.65832200	1.61888100	1.82052800
H	4.80929700	-2.01167000	0.86063200
H	4.43801900	-2.23836600	-0.84299400
H	6.73507900	-2.77148700	-0.43413400
H	7.10234800	-1.18768000	0.23984500
H	6.68736100	-1.35432400	-1.48091100

Computed SI coordinates of structure of DEAHF in $[BMIm][PF_6]$ in T form (S_1 state)

C	-3.72691100	-2.26780900	0.18888400
C	-3.19793600	-0.99438800	0.07984800
C	-4.03695100	0.14762200	0.01461600
C	-5.44101800	-0.05287900	0.06516900
C	-5.95994700	-1.32767200	0.17370300
C	-5.11146200	-2.43597200	0.23568600
C	-3.40764100	1.38765800	-0.09390900
C	-1.97123700	1.51912100	-0.14248600
C	-1.19226800	0.33062000	-0.06682900
O	-1.84634500	-0.88631800	0.03643500
C	0.22064100	0.20603400	-0.08820200
C	0.84384700	-1.07004600	-0.02638600

C	2.20882100	-1.21787000	-0.05673600
C	3.07769900	-0.09963600	-0.15895600
C	2.45582600	1.17965800	-0.18986000
C	1.09319000	1.32514100	-0.16073900
N	4.43350700	-0.24647700	-0.23203200
C	5.31496300	0.90875100	-0.11060900
C	5.56153600	1.33899800	1.33126500
C	5.00951900	-1.59131500	-0.12574200
C	6.47791900	-1.70135700	-0.48583600
O	-4.10254800	2.52699600	-0.16514200
O	-1.51333800	2.69999100	-0.24806600
H	-3.41321500	3.22455400	-0.23751400
H	-3.05472300	-3.11662800	0.23559700
H	-6.09374300	0.81026900	0.01682500
H	-7.03438400	-1.46950700	0.21089300
H	-5.52417800	-3.43472000	0.32053100
H	0.22979200	-1.95743100	0.05224300
H	2.61056800	-2.21923000	0.00827500
H	3.05627200	2.07742500	-0.23636500
H	0.66657600	2.31779700	-0.19262100
H	6.25984000	0.65897500	-0.58864300
H	4.89953400	1.73353100	-0.68942900
H	6.23037000	2.20372000	1.34729500
H	6.02993900	0.53783900	1.90774000
H	4.63002000	1.61931100	1.82795900
H	4.85274200	-1.97440600	0.89197300
H	4.44841000	-2.24447900	-0.79746900
H	6.75216200	-2.75805300	-0.44131700
H	7.12959700	-1.16592900	0.20655600
H	6.67617500	-1.35099400	-1.50140500

2. We employed the TZVP basis set along with mPW1PW91 functionals（with SMD）:

Computed SI coordinates of structure of DEAHF in [EMIm][NTf_2] in N form (S_0 state)

C	-3.74074200	-2.26412300	0.03619000
C	-3.20839900	-0.97710800	0.00825900
C	-4.03324300	0.14751600	0.03005100
C	-5.42167400	-0.03391900	0.07659700
C	-5.95603100	-1.30154300	0.10379600
C	-5.10974900	-2.41668200	0.08331300
C	-3.41438200	1.45784300	-0.00543700
C	-1.97348000	1.47373900	-0.03293600
C	-1.22922000	0.32884800	-0.06553800
O	-1.86318800	-0.87426700	-0.04464600
C	0.21486500	0.19595900	-0.09854600
C	0.82021300	-1.04225300	0.15681600
C	2.18728000	-1.20683900	0.12702000
C	3.05483400	-0.13509800	-0.18398400
C	2.43949200	1.11548200	-0.41672500
C	1.07207300	1.27028200	-0.38159500
N	4.41026400	-0.30563500	-0.26770600
C	5.27860256	0.77292089	-0.28662528
C	5.50390756	1.41857989	1.07262572
C	4.98645344	-1.52007689	0.09300128
C	6.44629044	-1.70717089	-0.26688072
O	-3.97313113	2.55806989	0.00672963
O	-1.42543000	2.70830600	-0.03056400
H	-2.25073487	3.28253211	-0.00636863
H	-3.07450000	-3.11688200	0.01915300

H	-6.05546500	0.84353200	0.09246100
H	-7.02894900	-1.44011100	0.14050000
H	-5.53206000	-3.41361600	0.10456300
H	0.20620800	-1.89932000	0.39811700
H	2.58259100	-2.18425300	0.35753900
H	3.03744800	1.98749900	-0.63527800
H	0.66149200	2.24931200	-0.57713600
H	6.23060856	0.45825689	-0.70699928
H	4.87761856	1.49992689	-0.99183628
H	6.17258456	2.27655289	0.97388072
H	5.96086356	0.71463789	1.77096072
H	4.56695756	1.76913689	1.50887472
H	4.85069644	-1.69729789	1.16791428
H	4.41767644	-2.29243089	-0.42658372
H	6.72173144	-2.73785689	-0.03671672
H	7.11225244	-1.05724289	0.30138028
H	6.62376744	-1.54602989	-1.33179272

Computed SI coordinates of structure of DEAHF in [EMIm][NTf$_2$] in T form (S_0 state)

C	-3.72652416	-2.25877122	0.18668571
C	-3.19054416	-0.97634222	0.07917771
C	-4.01344516	0.16161778	0.01036471
C	-5.41434316	-0.01733822	0.05555171
C	-5.94135616	-1.28149322	0.16173171
C	-5.09688216	-2.40425122	0.22789571
C	-3.35164516	1.39809078	-0.09395129
C	-1.93485916	1.52660078	-0.13920929
C	-1.19663816	0.31968778	-0.06233429
O	-1.85047516	-0.86226722	0.04118471

C	0.23225084	0.18579678	-0.08836229
C	0.85423984	-1.07651222	-0.01567029
C	2.22198184	-1.21684122	-0.04337029
C	3.08311084	-0.09482322	-0.16400729
C	2.45324784	1.17387178	-0.20439429
C	1.08387184	1.30620878	-0.17754629
N	4.43977684	-0.23662122	-0.24623629
C	5.30693919	0.89628840	-0.11346776
C	5.53513419	1.33835140	1.32676824
C	5.02699648	-1.54438184	-0.10181681
C	6.48761948	-1.65627984	-0.49637581
O	-3.99333316	2.55229478	-0.16512929
O	-1.50657832	2.72439146	-0.24034142
H	-3.22796968	3.15723754	-0.22962958
H	-3.06053916	-3.11201722	0.23663571
H	-6.05792416	0.85254578	0.00453971
H	-7.01611516	-1.41786722	0.19560871
H	-5.52585116	-3.39610122	0.31191671
H	0.24868684	-1.96961022	0.07571171
H	2.62918884	-2.21467722	0.03530571
H	3.04490484	2.07683778	-0.26027229
H	0.63841084	2.29121478	-0.22050329
H	6.25986419	0.65506140	-0.58052176
H	4.89321419	1.71484940	-0.70307176
H	6.19359919	2.21113540	1.34919524
H	6.00701819	0.54558140	1.91268524
H	4.59687019	1.61028340	1.81592124
H	4.89937548	-1.90000984	0.92972419
H	4.45968348	-2.22294784	-0.74178081
H	6.77152148	-2.70998984	-0.43958281

H	7.15413448	-1.10335084	0.16787319
H	6.65752948	-1.32217284	-1.52283181

Computed SI coordinates of structure of DEAHF in [EMIm][NTf$_2$] in N form (S$_1$ state)

C	-3.73592471	-2.25975500	0.17572067
C	-3.20163371	-0.99089200	0.07392267
C	-4.02883171	0.15361700	0.01681067
C	-5.42739271	-0.04199800	0.06766967
C	-5.95988571	-1.31373400	0.16882167
C	-5.12342171	-2.42548900	0.22320167
C	-3.42141771	1.43342200	-0.08410733
C	-1.96844271	1.45100500	-0.12764033
C	-1.19061071	0.31177300	-0.06223533
O	-1.84076971	-0.90427000	0.03097367
C	0.22920629	0.19196400	-0.08895833
C	0.84211129	-1.08796300	-0.04364533
C	2.20223929	-1.24121600	-0.07580833
C	3.07240829	-0.12154400	-0.15898833
C	2.45849729	1.16260000	-0.18166633
C	1.10029029	1.31080600	-0.14890633
N	4.42034429	-0.27234200	-0.21434333
C	5.31166641	0.91534654	-0.15883525
C	5.58693441	1.38872754	1.26214475
C	4.98729117	-1.65406655	-0.11991141
C	6.47149817	-1.75619355	-0.39240341
O	-3.98429003	2.56577218	-0.14675575
O	-1.47963671	2.68401000	-0.22968833
H	-2.35756897	3.22917382	-0.24208225
H	-3.06833171	-3.11113900	0.21692067

H	-6.06680071	0.83016500	0.02482567
H	-7.03474071	-1.44553200	0.20593867
H	-5.53936371	-3.42183600	0.30260367
H	0.21993329	-1.96860000	0.01944367
H	2.60238729	-2.24254100	-0.02889533
H	3.06245929	2.05633000	-0.21825833
H	0.68930729	2.30825300	-0.16894133
H	6.24323141	0.64102754	-0.64543325
H	4.88473541	1.71900354	-0.75529925
H	6.26243841	2.24576254	1.23377975
H	6.05845941	0.60275054	1.85370175
H	4.66725341	1.69301454	1.76356075
H	4.76951117	-2.05562055	0.87650159
H	4.46176617	-2.28636255	-0.83749741
H	6.74408617	-2.81051555	-0.32472341
H	7.07533517	-1.21433755	0.33537159
H	6.72936617	-1.41317955	-1.39524841

Computed SI coordinates of structure of DEAHF in [EMIm][NTf$_2$] in T form (S$_1$ state)

C	-3.72652416	-2.25877122	0.18668571
C	-3.19054416	-0.97634222	0.07917771
C	-4.01344516	0.16161778	0.01036471
C	-5.41434316	-0.01733822	0.05555171
C	-5.94135616	-1.28149322	0.16173171
C	-5.09688216	-2.40425122	0.22789571
C	-3.35164516	1.39809078	-0.09395129
C	-1.93485916	1.52660078	-0.13920929
C	-1.19663816	0.31968778	-0.06233429
O	-1.85047516	-0.86226722	0.04118471

C	0.23225084	0.18579678	-0.08836229
C	0.85423984	-1.07651222	-0.01567029
C	2.22198184	-1.21684122	-0.04337029
C	3.08311084	-0.09482322	-0.16400729
C	2.45324784	1.17387178	-0.20439429
C	1.08387184	1.30620878	-0.17754629
N	4.43977684	-0.23662122	-0.24623629
C	5.30693919	0.89628840	-0.11346776
C	5.53513419	1.33835140	1.32676824
C	5.02699648	-1.54438184	-0.10181681
C	6.48761948	-1.65627984	-0.49637581
O	-3.99333316	2.55229478	-0.16512929
O	-1.54086510	2.73301291	-0.24012806
H	-3.19368290	3.14861609	-0.22984294
H	-3.06053916	-3.11201722	0.23663571
H	-6.05792416	0.85254578	0.00453971
H	-7.01611516	-1.41786722	0.19560871
H	-5.52585116	-3.39610122	0.31191671
H	0.24868684	-1.96961022	0.07571171
H	2.62918884	-2.21467722	0.03530571
H	3.04490484	2.07683778	-0.26027229
H	0.63841084	2.29121478	-0.22050329
H	6.25986419	0.65506140	-0.58052176
H	4.89321419	1.71484940	-0.70307176
H	6.19359919	2.21113540	1.34919524
H	6.00701819	0.54558140	1.91268524
H	4.59687019	1.61028340	1.81592124
H	4.89937548	-1.90000984	0.92972419
H	4.45968348	-2.22294784	-0.74178081
H	6.77152148	-2.70998984	-0.43958281

H	7.15413448	-1.10335084	0.16787319
H	6.65752948	-1.32217284	-1.52283181

Computed SI coordinates of structure of DEAHF in [BMIm][PF_6] in N form (S_0 state)

C	-3.74074200	-2.26412300	0.03619000
C	-3.20839900	-0.97710800	0.00825900
C	-4.03324300	0.14751600	0.03005100
C	-5.42167400	-0.03391900	0.07659700
C	-5.95603100	-1.30154300	0.10379600
C	-5.10974900	-2.41668200	0.08331300
C	-3.41438200	1.45784300	-0.00543700
C	-1.97348000	1.47373900	-0.03293600
C	-1.22922000	0.32884800	-0.06553800
O	-1.86318800	-0.87426700	-0.04464600
C	0.21486500	0.19595900	-0.09854600
C	0.82021300	-1.04225300	0.15681600
C	2.18728000	-1.20683900	0.12702000
C	3.05483400	-0.13509800	-0.18398400
C	2.43949200	1.11548200	-0.41672500
C	1.07207300	1.27028200	-0.38159500
N	4.41026400	-0.30563500	-0.26770600
C	5.27860256	0.77292089	-0.28662528
C	5.50390756	1.41857989	1.07262572
C	4.98645344	-1.52007689	0.09300128
C	6.44629044	-1.70717089	-0.26688072
O	-3.97313113	2.55806989	0.00672963
O	-1.42543000	2.70830600	-0.03056400
H	-2.25073487	3.28253211	-0.00636863
H	-3.07450000	-3.11688200	0.01915300
H	-6.05546500	0.84353200	0.09246100

H	-7.02894900	-1.44011100	0.14050000
H	-5.53206000	-3.41361600	0.10456300
H	0.20620800	-1.89932000	0.39811700
H	2.58259100	-2.18425300	0.35753900
H	3.03744800	1.98749900	-0.63527800
H	0.66149200	2.24931200	-0.57713600
H	6.23060856	0.45825689	-0.70699928
H	4.87761856	1.49992689	-0.99183628
H	6.17258456	2.27655289	0.97388072
H	5.96086356	0.71463789	1.77096072
H	4.56695756	1.76913689	1.50887472
H	4.85069644	-1.69729789	1.16791428
H	4.41767644	-2.29243089	-0.42658372
H	6.72173144	-2.73785689	-0.03671672
H	7.11225244	-1.05724289	0.30138028
H	6.62376744	-1.54602989	-1.33179272

Computed SI coordinates of structure of DEAHF in [BMIm][PF_6] in T form (S_0 state)

C	-3.72621103	-2.25852372	0.18665932
C	-3.19023103	-0.97609472	0.07915132
C	-4.01313203	0.16186528	0.01033832
C	-5.41403003	-0.01709072	0.05552532
C	-5.94104303	-1.28124572	0.16170532
C	-5.09656903	-2.40400372	0.22786932
C	-3.35133203	1.39833828	-0.09397768
C	-1.93454603	1.52684828	-0.13923568
C	-1.19632503	0.31993528	-0.06236068
O	-1.85016203	-0.86201972	0.04115832
C	0.23256397	0.18604428	-0.08838868
C	0.85455297	-1.07626472	-0.01569668

C	2.22229497	-1.21659372	-0.04339668
C	3.08342397	-0.09457572	-0.16403368
C	2.45356097	1.17411928	-0.20442068
C	1.08418497	1.30645628	-0.17757268
N	4.44008997	-0.23637372	-0.24626268
C	5.30725232	0.89653590	-0.11349415
C	5.53544732	1.33859890	1.32674185
C	5.02730961	-1.54413434	-0.10184320
C	6.48793261	-1.65603234	-0.49640220
O	-3.99302003	2.55254228	-0.16515568
O	-1.50602105	2.72457766	-0.24036934
H	-3.22852695	3.15705134	-0.22960166
H	-3.06022603	-3.11176972	0.23660932
H	-6.05761103	0.85279328	0.00451332
H	-7.01580203	-1.41761972	0.19558232
H	-5.52553803	-3.39585372	0.31189032
H	0.24899997	-1.96936272	0.07568532
H	2.62950197	-2.21442972	0.03527932
H	3.04521797	2.07708528	-0.26029868
H	0.63872397	2.29146228	-0.22052968
H	6.26017732	0.65530890	-0.58054815
H	4.89352732	1.71509690	-0.70309815
H	6.19391232	2.21138290	1.34916885
H	6.00733132	0.54582890	1.91265885
H	4.59718332	1.61053090	1.81589485
H	4.89968861	-1.89976234	0.92969780
H	4.45999661	-2.22270034	-0.74180720
H	6.77183461	-2.70974234	-0.43960920
H	7.15444761	-1.10310334	0.16784680
H	6.65784261	-1.32192534	-1.52285820

Computed SI coordinates of structure of DEAHF in [BMIm][PF_6] in N form (S_1 state)

C	-3.73644345	-2.25983540	0.17574180
C	-3.20215245	-0.99097240	0.07394380
C	-4.02935045	0.15353660	0.01683180
C	-5.42791145	-0.04207840	0.06769080
C	-5.96040445	-1.31381440	0.16884280
C	-5.12394045	-2.42556940	0.22322280
C	-3.42193645	1.43334160	-0.08408620
C	-1.96896145	1.45092460	-0.12761920
C	-1.19112945	0.31169260	-0.06221420
O	-1.84128845	-0.90435040	0.03099480
C	0.22868755	0.19188360	-0.08893720
C	0.84159255	-1.08804340	-0.04362420
C	2.20172055	-1.24129640	-0.07578720
C	3.07188955	-0.12162440	-0.15896720
C	2.45797855	1.16251960	-0.18164520
C	1.09977155	1.31072560	-0.14888520
N	4.41982555	-0.27242240	-0.21432220
C	5.31114767	0.91526614	-0.15881412
C	5.58641567	1.38864714	1.26216588
C	4.98677243	-1.65414695	-0.11989028
C	6.47097943	-1.75627395	-0.39238228
O	-3.98480877	2.56569178	-0.14673462
O	-1.48015545	2.68392960	-0.22966720
H	-2.35705023	3.22925422	-0.24210338
H	-3.06885045	-3.11121940	0.21694180
H	-6.06731945	0.83008460	0.02484680
H	-7.03525945	-1.44561240	0.20595980
H	-5.53988245	-3.42191640	0.30262480

H	0.21941455	-1.96868040	0.01946480
H	2.60186855	-2.24262140	-0.02887420
H	3.06194055	2.05624960	-0.21823720
H	0.68878855	2.30817260	-0.16892020
H	6.24271267	0.64094714	-0.64541212
H	4.88421667	1.71892314	-0.75527812
H	6.26191967	2.24568214	1.23380088
H	6.05794067	0.60267014	1.85372288
H	4.66673467	1.69293414	1.76358188
H	4.76899243	-2.05570095	0.87652272
H	4.46124743	-2.28644295	-0.83747628
H	6.74356743	-2.81059595	-0.32470228
H	7.07481643	-1.21441795	0.33539272
H	6.72884743	-1.41325995	-1.39522728

Computed SI coordinates of structure of DEAHF in [BMIm][PF_6] in T form (S_1 state)

C	-3.72671868	-2.25891628	0.18670145
C	-3.19073868	-0.97648728	0.07919345
C	-4.01363968	0.16147272	0.01038045
C	-5.41453768	-0.01748328	0.05556745
C	-5.94155068	-1.28163828	0.16174745
C	-5.09707668	-2.40439628	0.22791145
C	-3.35183968	1.39794572	-0.09393555
C	-1.93505368	1.52645572	-0.13919355
C	-1.19683268	0.31954272	-0.06231855
O	-1.85066968	-0.86241228	0.04120045
C	0.23205632	0.18565172	-0.08834655
C	0.85404532	-1.07665728	-0.01565455
C	2.22178732	-1.21698628	-0.04335455
C	3.08291632	-0.09496828	-0.16399155

C	2.45305332	1.17372672	-0.20437855
C	1.08367732	1.30606372	-0.17753055
N	4.43958232	-0.23676628	-0.24622055
C	5.30674467	0.89614334	-0.11345202
C	5.53493967	1.33820634	1.32678398
C	5.02680196	-1.54452690	-0.10180107
C	6.48742496	-1.65642490	-0.49636007
O	-3.99352768	2.55214972	-0.16511355
O	-1.54105962	2.73286785	-0.24011232
H	-3.19348838	3.14876115	-0.22985868
H	-3.06073368	-3.11216228	0.23665145
H	-6.05811868	0.85240072	0.00455545
H	-7.01630968	-1.41801228	0.19562445
H	-5.52604568	-3.39624628	0.31193245
H	0.24849232	-1.96975528	0.07572745
H	2.62899432	-2.21482228	0.03532145
H	3.04471032	2.07669272	-0.26025655
H	0.63821632	2.29106972	-0.22048755
H	6.25966967	0.65491634	-0.58050602
H	4.89301967	1.71470434	-0.70305602
H	6.19340467	2.21099034	1.34921098
H	6.00682367	0.54543634	1.91270098
H	4.59667567	1.61013834	1.81593698
H	4.89918096	-1.90015490	0.92973993
H	4.45948896	-2.22309290	-0.74176507
H	6.77132696	-2.71013490	-0.43956707
H	7.15393996	-1.10349590	0.16788893
H	6.65733496	-1.32231790	-1.52281607

3. We employed the TZVP basis set along with CAM-B3LYP functionals（with SMD）:

Computed SI coordinates of structure of DEAHF in [EMIm][NTf_2] in N form (S_0 state)

C	-3.75131600	-2.26161600	0.00087500
C	-3.21541100	-0.97658000	-0.00769700
C	-4.03349900	0.14666900	0.03425900
C	-5.42174900	-0.02908500	0.08252300
C	-5.95972700	-1.29227600	0.09081800
C	-5.11757200	-2.40963700	0.04993300
C	-3.41056100	1.45821200	0.02099300
C	-1.96681100	1.47002900	-0.00984100
C	-1.23296100	0.32970300	-0.06492000
O	-1.86718100	-0.87499500	-0.06398300
C	0.21790900	0.19409700	-0.10432500
C	0.82091600	-1.03001000	0.19514700
C	2.18764900	-1.19665700	0.16123300
C	3.05060200	-0.14101600	-0.20003500
C	2.43518100	1.09686600	-0.47906400
C	1.06834800	1.25362400	-0.43799400
N	4.41087600	-0.31583400	-0.28795900
C	5.29009700	0.84761800	-0.33868800
C	5.50993500	1.51476600	1.01221300
C	4.97814200	-1.59177800	0.15678000
C	6.44001800	-1.79607300	-0.19260600
O	-4.02093700	2.53162600	0.05420000
O	-1.40578700	2.70472200	0.01476600
H	-2.16248000	3.32027900	0.05546700
H	-3.08705100	-3.11489700	-0.03207100
H	-6.05197000	0.84977700	0.11443600

H	-7.03242100	-1.42789500	0.12871600
H	-5.54420800	-3.40441800	0.05661100
H	0.20755400	-1.87566600	0.47339200
H	2.58295100	-2.16405300	0.42619400
H	3.03048100	1.95807500	-0.73867000
H	0.65488800	2.22307600	-0.66937200
H	6.24354400	0.52905400	-0.74991800
H	4.89172300	1.56247700	-1.05547600
H	6.18024800	2.36993500	0.90507100
H	5.96184400	0.82014200	1.72269700
H	4.57146900	1.87249800	1.43822700
H	4.84222900	-1.71193100	1.23903400
H	4.41328900	-2.39128100	-0.32116400
H	6.71644800	-2.81459800	0.08375400
H	7.10473100	-1.11998300	0.34454100
H	6.61669400	-1.68170200	-1.26326400

Computed SI coordinates of structure of DEAHF in [EMIm][NTf_2] in T form (S_0 state)

C	-4.01380062	-2.38816025	0.26144281
C	-3.28668211	-1.24139569	-0.08189915
C	-3.92344252	0.00405325	-0.05190636
C	-5.29596323	0.07480961	0.21329709
C	-5.98833929	-1.05306532	0.50846327
C	-5.33615012	-2.29550384	0.55092387
C	-3.19406495	1.17240478	-0.27165135
C	-1.80570807	1.09892778	-0.38480282
C	-1.16448133	-0.14721152	-0.37458024
O	-1.91028705	-1.37060243	-0.47255343
C	0.23262541	-0.20032837	-0.27869813
C	0.88800619	-1.43902432	-0.27318571

C	2.23905473	-1.49038990	-0.18046861
C	2.98078095	-0.30481067	-0.09010287
C	2.32540001	0.93388515	-0.09561216
C	0.97435140	0.98525074	-0.18832839
N	4.31438326	-0.35551287	0.00141410
C	5.09241758	0.88810163	0.09620617
C	5.26298016	1.27530714	1.57694256
C	5.00184599	-1.65484407	0.00719016
C	6.40045156	-1.49839639	-0.61813091
O	-3.80029674	2.32120129	-0.34995379
O	-1.12714746	2.15388670	-0.48573743
H	-3.14601273	3.35438959	-0.53530746
H	-3.52661100	-3.34038061	0.29012455
H	-5.79769855	1.01955834	0.18856784
H	-7.03704103	-0.99540661	0.71291403
H	-5.88622103	-3.17579385	0.81056110
H	0.32168204	-2.34424028	-0.34218579
H	2.73945271	-2.43616163	-0.17626267
H	2.89172441	1.83910114	-0.02661443
H	0.47395368	1.93102260	-0.19253744
H	6.05507351	0.74123424	-0.34721633
H	4.57757821	1.67115262	-0.42019636
H	5.82930449	2.18052311	1.64594086
H	5.77781953	0.49225617	2.09334504
H	4.30032422	1.42217455	2.02036512
H	5.09837833	-2.00280755	1.01441551
H	4.43395328	-2.36135296	-0.56135452
H	6.90084958	-2.44416810	-0.61392658
H	6.96834429	-0.79188751	-0.04958622
H	6.30391922	-1.15043289	-1.62535623

Computed SI coordinates of structure of DEAHF in [EMIm][NTf_2] in N form (S_1 state)

C	-3.70988600	-2.25787800	0.33151400
C	-3.19519400	-0.97733800	0.14777800
C	-4.03248900	0.12453100	0.01476500
C	-5.41816000	-0.06826400	0.07086500
C	-5.93526600	-1.32718700	0.25197200
C	-5.07412800	-2.42304700	0.38204800
C	-3.43157600	1.43278200	-0.17438100
C	-1.98885100	1.46100400	-0.22943100
C	-1.23457800	0.34322100	-0.07651400
O	-1.84768200	-0.85773800	0.11058200
C	0.21807200	0.22845700	-0.11147000
C	0.82458200	-1.01735600	-0.28946800
C	2.19376800	-1.16303900	-0.32286400
C	3.05677100	-0.05483000	-0.19154300
C	2.43664300	1.19403800	0.02252400
C	1.06719000	1.32850600	0.04989200
N	4.42125600	-0.18597800	-0.27978100
C	5.28472700	0.90620200	0.15556000
C	5.44432700	1.00459700	1.66654000
C	4.98833000	-1.53551200	-0.35160300
C	6.47076600	-1.58918200	-0.66782600
O	-4.06032400	2.48772400	-0.30804000
O	-1.44942200	2.68712000	-0.44236500
H	-2.21661200	3.28605700	-0.51722200
H	-3.03093500	-3.09417900	0.43140900
H	-6.06322000	0.79411900	-0.03307400
H	-7.00603300	-1.47610300	0.29430000

H	-5.48431800	-3.41460500	0.52436000
H	0.21148200	-1.90099200	-0.39915400
H	2.59041300	-2.15706900	-0.45377600
H	3.02996500	2.08247000	0.17219300
H	0.65025600	2.31085700	0.20988300
H	6.25776300	0.76944200	-0.30713000
H	4.90150700	1.84077100	-0.24857600
H	6.10198500	1.83795800	1.92185700
H	5.88397000	0.09211700	2.07345500
H	4.48539200	1.16781300	2.16038100
H	4.79617500	-2.07776800	0.58276900
H	4.46585300	-2.07498500	-1.14102600
H	6.75208600	-2.63496200	-0.79953800
H	7.09078100	-1.18167100	0.13033000
H	6.70381400	-1.06249800	-1.59453700

Computed SI coordinates of structure of DEAHF in [EMIm][NTf$_2$] in T form (S$_1$ state)

C	-3.73180700	-2.26164600	0.17486700
C	-3.19642800	-0.99623200	0.07545800
C	-4.02459100	0.14828500	0.01639800
C	-5.42425000	-0.04301400	0.06681300
C	-5.95233000	-1.31074300	0.16726300
C	-5.11262100	-2.42167000	0.22241800
C	-3.39460200	1.38838800	-0.08023900
C	-1.96852700	1.51342500	-0.13824200
C	-1.18091300	0.31759700	-0.06824600
O	-1.84111000	-0.89588400	0.03016400
C	0.21591300	0.20186600	-0.08584200
C	0.84378300	-1.07494000	-0.02315100

C	2.20108300	-1.22106600	-0.05330500
C	3.07091100	-0.10235500	-0.15969700
C	2.44731900	1.17745200	-0.18642000
C	1.09259700	1.32292300	-0.15705500
N	4.41826600	-0.24962100	-0.23478100
C	5.30278600	0.90905400	-0.14352900
C	5.56167600	1.35714500	1.28807800
C	4.99598100	-1.59469500	-0.10795000
C	6.47040400	-1.70094200	-0.44154500
O	-4.09852900	2.52714500	-0.14497700
O	-1.49651000	2.68012900	-0.24361700
H	-3.42576100	3.23528000	-0.22073400
H	-3.06591300	-3.11365700	0.21609700
H	-6.06922400	0.82412300	0.02374200
H	-7.02577400	-1.44503700	0.20434500
H	-5.53087700	-3.41667800	0.30115600
H	0.22899600	-1.95938600	0.05652100
H	2.60376800	-2.21955800	0.01063600
H	3.04784500	2.07256600	-0.22794800
H	0.66488100	2.31286500	-0.18572700
H	6.24061300	0.65332000	-0.62617200
H	4.88185500	1.72258100	-0.72936100
H	6.23302900	2.21788000	1.29141500
H	6.02942500	0.56070000	1.86891700
H	4.63598600	1.64564500	1.78751100
H	4.82440000	-1.96677500	0.90839600
H	4.45230700	-2.25458800	-0.78328700
H	6.75136700	-2.75278700	-0.37421300
H	7.10394800	-1.14748400	0.25074300
H	6.68216100	-1.36577300	-1.45756400

Computed SI coordinates of structure of DEAHF in [BMIm][PF_6] in N form (S_0 state)

C	-3.75081014	-2.26202751	0.00084779
C	-3.21490514	-0.97699151	-0.00772421
C	-4.03299314	0.14625749	0.03423179
C	-5.42124314	-0.02949651	0.08249579
C	-5.95922114	-1.29268751	0.09079079
C	-5.11706614	-2.41004851	0.04990579
C	-3.41005514	1.45780049	0.02096579
C	-1.96630514	1.46961749	-0.00986821
C	-1.23245514	0.32929149	-0.06494721
O	-1.86667514	-0.87540651	-0.06401021
C	0.21841486	0.19368549	-0.10435221
C	0.82142186	-1.03042151	0.19511979
C	2.18815486	-1.19706851	0.16120579
C	3.05110786	-0.14142751	-0.20006221
C	2.43568686	1.09645449	-0.47909121
C	1.06885386	1.25321249	-0.43802121
N	4.41138186	-0.31624551	-0.28798621
C	5.29060286	0.84720649	-0.33871521
C	5.51044086	1.51435449	1.01218579
C	4.97864786	-1.59218951	0.15675279
C	6.44052386	-1.79648451	-0.19263321
O	-4.01741211	2.52372611	0.05378896
O	-1.40759663	2.70121732	0.01464434
H	-2.16256080	3.32391956	0.05562865
H	-3.08654514	-3.11530851	-0.03209821
H	-6.05146414	0.84936549	0.11440879
H	-7.03191514	-1.42830651	0.12868879
H	-5.54370214	-3.40482951	0.05658379

H	0.20805986	-1.87607751	0.47336479
H	2.58345686	-2.16446451	0.42616679
H	3.03098686	1.95766349	-0.73869721
H	0.65539386	2.22266449	-0.66939921
H	6.24404986	0.52864249	-0.74994521
H	4.89222886	1.56206549	-1.05550321
H	6.18075386	2.36952349	0.90504379
H	5.96234986	0.81973049	1.72266979
H	4.57197486	1.87208649	1.43819979
H	4.84273486	-1.71234251	1.23900679
H	4.41379486	-2.39169251	-0.32119121
H	6.71695386	-2.81500951	0.08372679
H	7.10523686	-1.12039451	0.34451379
H	6.61719986	-1.68211351	-1.26329121

Computed SI coordinates of structure of DEAHF in [BMIm][PF_6] in T form (S_0 state)

C	-4.01380062	-2.38816025	0.26144281
C	-3.28668211	-1.24139569	-0.08189915
C	-3.92344252	0.00405325	-0.05190636
C	-5.29596323	0.07480961	0.21329709
C	-5.98833929	-1.05306532	0.50846327
C	-5.33615012	-2.29550384	0.55092387
C	-3.19406495	1.17240478	-0.27165135
C	-1.80570807	1.09892778	-0.38480282
C	-1.16448133	-0.14721152	-0.37458024
O	-1.91028705	-1.37060243	-0.47255343
C	0.23262541	-0.20032837	-0.27869813
C	0.88800619	-1.43902432	-0.27318571
C	2.23905473	-1.49038990	-0.18046861
C	2.98078095	-0.30481067	-0.09010287

C	2.32540001	0.93388515	-0.09561216
C	0.97435140	0.98525074	-0.18832839
N	4.31438326	-0.35551287	0.00141410
C	5.09241758	0.88810163	0.09620617
C	5.26298016	1.27530714	1.57694256
C	5.00184599	-1.65484407	0.00719016
C	6.40045156	-1.49839639	-0.61813091
O	-3.80033518	2.32122543	-0.34995492
O	-1.12710365	2.15395985	-0.48575040
H	-3.14600290	3.35434954	-0.53530237
H	-3.52661100	-3.34038061	0.29012455
H	-5.79769855	1.01955834	0.18856784
H	-7.03704103	-0.99540661	0.71291403
H	-5.88622103	-3.17579385	0.81056110
H	0.32168204	-2.34424028	-0.34218579
H	2.73945271	-2.43616163	-0.17626267
H	2.89172441	1.83910114	-0.02661443
H	0.47395368	1.93102260	-0.19253744
H	6.05507351	0.74123424	-0.34721633
H	4.57757821	1.67115262	-0.42019636
H	5.82930449	2.18052311	1.64594086
H	5.77781953	0.49225617	2.09334504
H	4.30032422	1.42217455	2.02036512
H	5.09837833	-2.00280755	1.01441551
H	4.43395328	-2.36135296	-0.56135452
H	6.90084958	-2.44416810	-0.61392658
H	6.96834429	-0.79188751	-0.04958622
H	6.30391922	-1.15043289	-1.62535623

Computed SI coordinates of structure of DEAHF in [BMIm][PF_6] in N form (S_1 state)

C	-3.70175200	-2.25981200	0.33720600
C	-3.19072200	-0.97867300	0.14966700
C	-4.02955600	0.12194800	0.01833000
C	-5.41484200	-0.07329800	0.07865500
C	-5.92867900	-1.33286000	0.26327500
C	-5.06536800	-2.42716800	0.39240500
C	-3.43177300	1.43015900	-0.17571100
C	-1.99124000	1.46249600	-0.23523400
C	-1.23436600	0.34561400	-0.08279700
O	-1.84353800	-0.85586300	0.10795700
C	0.21802000	0.23373300	-0.12055100
C	0.82471900	-1.00871000	-0.31961800
C	2.19383900	-1.15340400	-0.35301000
C	3.05588800	-0.04756700	-0.19981500
C	2.43547400	1.19734000	0.03409600
C	1.06608200	1.33117100	0.06119000
N	4.42115000	-0.17682700	-0.28738700
C	5.28095800	0.90491100	0.18092600
C	5.42523400	0.97035800	1.69488200
C	4.98736100	-1.52649000	-0.36674700
C	6.47293500	-1.57867100	-0.66698600
O	-4.06810700	2.48276400	-0.30992000
O	-1.44784100	2.68737600	-0.45379700
H	-2.20742200	3.29367000	-0.53695900
H	-3.01988200	-3.09383000	0.43619800
H	-6.06271700	0.78700400	-0.02484400
H	-6.99902100	-1.48361100	0.30902300
H	-5.47363300	-3.41905800	0.53769700
H	0.21173200	-1.89015200	-0.44715300
H	2.59099000	-2.14474900	-0.50132600

H	3.02837100	2.08306700	0.20036500
H	0.64899100	2.31027000	0.23980500
H	6.25835100	0.77782300	-0.27537800
H	4.90264400	1.84831700	-0.20683300
H	6.08027100	1.79784300	1.97470400
H	5.86069800	0.04935700	2.08678100
H	4.46141700	1.12314400	2.18251300
H	4.78415200	-2.07820700	0.55970800
H	4.47338600	-2.05718700	-1.16751600
H	6.75427500	-2.62324500	-0.80784100
H	7.08480700	-1.18170900	0.14267800
H	6.71715300	-1.04162700	-1.58480300

Computed SI coordinates of structure of DEAHF in [BMIm][PF_6] in T form (S_1 state)

C	-3.72592400	-2.26385500	0.17948800
C	-3.19321200	-0.99756200	0.07495300
C	-4.02236200	0.14538200	0.02254100
C	-5.42077700	-0.04684900	0.08721600
C	-5.94680100	-1.31484000	0.19338500
C	-5.10551000	-2.42493700	0.24048000
C	-3.39534000	1.38732900	-0.08311200
C	-1.97107400	1.51463900	-0.15832000
C	-1.18081700	0.32023300	-0.08311200
O	-1.83842800	-0.89563300	0.01596400
C	0.21600000	0.20639700	-0.09620100
C	0.84403400	-1.07153400	-0.05044200
C	2.20109000	-1.21721100	-0.07873900
C	3.07127300	-0.09696900	-0.16357300
C	2.44796600	1.18315500	-0.17030200
C	1.09341000	1.32832500	-0.14335300

N	4.41882100	-0.24300500	-0.23718900
C	5.30268700	0.91323400	-0.11263900
C	5.54971700	1.32859100	1.33059100
C	4.99628400	-1.58952900	-0.12522400
C	6.47209300	-1.69068100	-0.45334400
O	-4.10944900	2.52119900	-0.14827400
O	-1.49760600	2.68026300	-0.27996800
H	-3.45143000	3.24070300	-0.23265200
H	-3.05818700	-3.11472400	0.21398800
H	-6.06696500	0.81963900	0.04995300
H	-7.01965000	-1.45006200	0.24118400
H	-5.52220400	-3.42027100	0.32310800
H	0.22941400	-1.95744200	0.01202400
H	2.60322000	-2.21677700	-0.03031100
H	3.04849400	2.07894600	-0.19226600
H	0.66800900	2.31933600	-0.15513200
H	6.24430800	0.66840200	-0.59358100
H	4.88688100	1.73978300	-0.68359900
H	6.22023900	2.18947300	1.35867700
H	6.01359000	0.51972800	1.89716500
H	4.61983100	1.60506300	1.82902200
H	4.81995500	-1.97533700	0.88516600
H	4.45632600	-2.24033700	-0.81211200
H	6.75240500	-2.74359600	-0.40234700
H	7.10269500	-1.14936400	0.25110700
H	6.68877200	-1.33868600	-1.46259600

4. We employed the TZVP basis set along with WB97XD functionals （with SMD）:

Computed SI coordinates of structure of DEAHF in [EMIm][NTf_2] in N form (S_0

state)

C	-3.76506700	-2.25286000	-0.09014300
C	-3.21909700	-0.97035200	-0.04691200
C	-4.03030000	0.15750200	0.04516600
C	-5.42119400	-0.01124800	0.09256600
C	-5.96861100	-1.27146300	0.05004500
C	-5.13359400	-2.39318500	-0.04135000
C	-3.39930700	1.46766400	0.08827600
C	-1.95208200	1.46682300	0.04526900
C	-1.23454400	0.31863400	-0.06063200
O	-1.87316700	-0.87902100	-0.10650500
C	0.21836000	0.17510800	-0.11114800
C	0.82196000	-1.02540900	0.27172000
C	2.19013700	-1.19640200	0.23056100
C	3.04733800	-0.17116700	-0.22430200
C	2.42894800	1.04296100	-0.59288500
C	1.06118800	1.20513700	-0.54070300
N	4.40826800	-0.35418500	-0.31734500
C	5.27771100	0.80714500	-0.46739600
C	5.47907600	1.59126300	0.82363300
C	4.97926500	-1.56487800	0.27905400
C	6.45890000	-1.77198100	0.00964300
O	-4.00147000	2.54061600	0.17798300
O	-1.36998500	2.68565300	0.12732300
H	-2.11095800	3.31039100	0.20664900
H	-3.10721700	-3.10935800	-0.16207100
H	-6.04656600	0.86976100	0.16409500
H	-7.04262600	-1.40094400	0.08682000
H	-5.56725200	-3.38498000	-0.07488800
H	0.21107000	-1.84647100	0.62428800

H	2.58833700	-2.14299700	0.56238100
H	3.02030200	1.88154600	-0.92852900
H	0.64340100	2.15463100	-0.84333100
H	6.23791800	0.46247500	-0.84338100
H	4.87854700	1.45165600	-1.24915500
H	6.14007700	2.44316100	0.64975200
H	5.93238100	0.96097800	1.59228800
H	4.53009800	1.96951900	1.20944300
H	4.80391400	-1.57113500	1.36335100
H	4.44818100	-2.42400800	-0.13300500
H	6.74276600	-2.74772200	0.40794800
H	7.08600700	-1.02362500	0.49601900
H	6.67618600	-1.77131400	-1.06042400

Computed SI coordinates of structure of DEAHF in [EMIm][NTf$_2$] in T form (S_0 state)

C	-3.69658439	-2.24254866	0.15376138
C	-3.16694639	-0.97215266	0.06153238
C	-4.00041839	0.17081234	0.03138838
C	-5.39842539	-0.02594966	0.11059038
C	-5.92166639	-1.29862066	0.20595838
C	-5.07685739	-2.40790666	0.22945038
C	-3.37538139	1.41813134	-0.06142362
C	-1.95081539	1.54244034	-0.15583462
C	-1.16020539	0.34068634	-0.10267862
O	-1.81449839	-0.87154166	-0.01282462
C	0.23706061	0.22745734	-0.12248262
C	0.86472661	-1.05302266	-0.09072062
C	2.22326161	-1.19948066	-0.12054862
C	3.09425461	-0.07720466	-0.19337562

C	2.47183561	1.20453034	-0.19571062
C	1.11571061	1.35070134	-0.16454362
N	4.44235761	-0.22445066	-0.25864962
C	5.32146461	0.93474134	-0.14548462
C	5.54215361	1.37654534	1.29584838
C	5.01465861	-1.56847366	-0.10576362
C	6.51165361	-1.66608666	-0.32976862
O	-4.05426668	2.51874930	-0.10691573
O	-1.50320257	2.60274286	-0.26295536
H	-3.48464115	3.25465920	-0.18892760
H	-3.02741439	-3.09356266	0.17013338
H	-6.04859239	0.83909334	0.09176138
H	-6.99425539	-1.43623566	0.26472238
H	-5.49000639	-3.40606066	0.30359638
H	0.25038861	-1.94096766	-0.03838462
H	2.62434961	-2.20124666	-0.08539762
H	3.07269861	2.10158634	-0.21042262
H	0.69063361	2.34320434	-0.16851962
H	6.27246261	0.68294734	-0.60644862
H	4.91301561	1.74927734	-0.74090462
H	6.20917561	2.24068734	1.32274338
H	5.99771761	0.57459834	1.88045838
H	4.59991261	1.65534934	1.77155438
H	4.77301061	-1.94878366	0.89397438
H	4.52428161	-2.22574666	-0.82585462
H	6.79233161	-2.71660366	-0.23710562
H	7.08595261	-1.10531266	0.40869938
H	6.79672761	-1.33303366	-1.32917262

Computed SI coordinates of structure of DEAHF in [EMIm][NTf_2] in N form (S_1

state)

C	-3.69360400	-2.25981400	0.37037800
C	-3.18442900	-0.97767800	0.16658100
C	-4.02862200	0.11969400	0.01842200
C	-5.41489700	-0.07969400	0.07908900
C	-5.92611300	-1.33980500	0.27997700
C	-5.05846400	-2.43065400	0.42509300
C	-3.43609900	1.43113700	-0.19526100
C	-1.98961500	1.46150400	-0.25115400
C	-1.23724000	0.34350100	-0.08198000
O	-1.84009600	-0.85511300	0.12767400
C	0.21903200	0.23595800	-0.12303700
C	0.82953000	-0.98724600	-0.41127400
C	2.20181300	-1.12275000	-0.45164800
C	3.05808400	-0.02576400	-0.21385500
C	2.43163000	1.19726600	0.11038700
C	1.05950900	1.32186800	0.14317700
N	4.42578700	-0.14264400	-0.30708300
C	5.27367500	0.90105000	0.25679000
C	5.38531900	0.84490900	1.77554000
C	4.99185200	-1.48663100	-0.44956500
C	6.49048900	-1.52635800	-0.68908300
O	-4.07050500	2.47802600	-0.34915100
O	-1.44453200	2.67603000	-0.49338800
H	-2.20402200	3.27630300	-0.58323200
H	-3.01074200	-3.09218900	0.48169600
H	-6.06573900	0.77782700	-0.03715600
H	-6.99663700	-1.49315800	0.32592500
H	-5.46371200	-3.42255400	0.58255700
H	0.22041500	-1.86018500	-0.60738500

H	2.60381300	-2.09955000	-0.67338800
H	3.01909300	2.07167500	0.34724000
H	0.63562800	2.28350400	0.39494500
H	6.26107400	0.81122800	-0.18937200
H	4.90093700	1.87234100	-0.06476100
H	6.03439800	1.64551700	2.13702800
H	5.81064400	-0.10858200	2.09729400
H	4.40805200	0.95881100	2.24932000
H	4.74995700	-2.09422300	0.43291600
H	4.51139900	-1.96381600	-1.30494700
H	6.77485600	-2.56218600	-0.88277800
H	7.06586300	-1.18352700	0.17192200
H	6.77418500	-0.93321600	-1.56078900

Computed SI coordinates of structure of DEAHF in [EMIm][NTf_2] in T form (S_1 state)

C	-3.71839400	-2.26612700	0.15659900
C	-3.18875600	-0.99573100	0.06437000
C	-4.02222800	0.14723400	0.03422600
C	-5.42023500	-0.04952800	0.11342800
C	-5.94347600	-1.32219900	0.20879600
C	-5.09866700	-2.43148500	0.23228800
C	-3.39719100	1.39455300	-0.05858600
C	-1.97262500	1.51886200	-0.15299700
C	-1.18201500	0.31710800	-0.09984100
O	-1.83630800	-0.89512000	-0.00998700
C	0.21525100	0.20387900	-0.11964500
C	0.84291700	-1.07660100	-0.08788300
C	2.20145200	-1.22305900	-0.11771100
C	3.07244500	-0.10078300	-0.19053800
C	2.45002600	1.18095200	-0.19287300

C	1.09390100	1.32712300	-0.16170600
N	4.42054800	-0.24802900	-0.25581200
C	5.29965500	0.91116300	-0.14264700
C	5.52034400	1.35296700	1.29868600
C	4.99284900	-1.59205200	-0.10292600
C	6.48984400	-1.68966500	-0.32693100
O	-4.11100600	2.52641100	-0.10407200
O	-1.49679100	2.68148800	-0.26851300
H	-3.45138800	3.23952300	-0.18989400
H	-3.04922400	-3.11714100	0.17297100
H	-6.07040200	0.81551500	0.09459900
H	-7.01606500	-1.45981400	0.26756000
H	-5.51181600	-3.42963900	0.30643400
H	0.22857900	-1.96454600	-0.03554700
H	2.60254000	-2.22482500	-0.08256000
H	3.05088900	2.07800800	-0.20758500
H	0.66882400	2.31962600	-0.16568200
H	6.25065300	0.65936900	-0.60361100
H	4.89120600	1.72569900	-0.73806700
H	6.18736600	2.21710900	1.32558100
H	5.97590800	0.55102000	1.88329600
H	4.57810300	1.63177100	1.77439200
H	4.75120100	-1.97236200	0.89681200
H	4.50247200	-2.24932500	-0.82301700
H	6.77052200	-2.74018200	-0.23426800
H	7.06414300	-1.12889100	0.41153700
H	6.77491800	-1.35661200	-1.32633500

Computed SI coordinates of structure of DEAHF in [BMIm][PF_6] in N form (S_0 state)

C	-3.76534729	-2.25262368	-0.09011299
C	-3.21937729	-0.97011568	-0.04688199

C	-4.03058029	0.15773832	0.04519601
C	-5.42147429	-0.01101168	0.09259601
C	-5.96889129	-1.27122668	0.05007501
C	-5.13387429	-2.39294868	-0.04131999
C	-3.39958729	1.46790032	0.08830601
C	-1.95236229	1.46705932	0.04529901
C	-1.23482429	0.31887032	-0.06060199
O	-1.87344729	-0.87878468	-0.10647499
C	0.21807971	0.17534432	-0.11111799
C	0.82167971	-1.02517268	0.27175001
C	2.18985671	-1.19616568	0.23059101
C	3.04705771	-0.17093068	-0.22427199
C	2.42866771	1.04319732	-0.59285499
C	1.06090771	1.20537332	-0.54067299
N	4.40798771	-0.35394868	-0.31731499
C	5.27743071	0.80738132	-0.46736599
C	5.47879571	1.59149932	0.82366301
C	4.97898471	-1.56464168	0.27908401
C	6.45861971	-1.77174468	0.00967301
O	-4.00116035	2.54094692	0.17800772
O	-1.37030698	2.68584030	0.12734924
H	-2.11120865	3.30998560	0.20661555
H	-3.10749729	-3.10912168	-0.16204099
H	-6.04684629	0.86999732	0.16412501
H	-7.04290629	-1.40070768	0.08685001
H	-5.56753229	-3.38474368	-0.07485799
H	0.21078971	-1.84623468	0.62431801
H	2.58805671	-2.14276068	0.56241101
H	3.02002171	1.88178232	-0.92849899
H	0.64312071	2.15486732	-0.84330099

H	6.23763771	0.46271132	-0.84335099
H	4.87826671	1.45189232	-1.24912499
H	6.13979671	2.44339732	0.64978201
H	5.93210071	0.96121432	1.59231801
H	4.52981771	1.96975532	1.20947301
H	4.80363371	-1.57089868	1.36338101
H	4.44790071	-2.42377168	-0.13297499
H	6.74248571	-2.74748568	0.40797801
H	7.08572671	-1.02338868	0.49604901
H	6.67590571	-1.77107768	-1.06039399

Computed SI coordinates of structure of DEAHF in [BMIm][PF_6] in T form (S_0 state)

C	-3.69484127	-2.24029669	0.15351041
C	-3.16520327	-0.96990069	0.06128141
C	-3.99867527	0.17306431	0.03113741
C	-5.39668227	-0.02369769	0.11033941
C	-5.91992327	-1.29636869	0.20570741
C	-5.07511427	-2.40565469	0.22919941
C	-3.37363827	1.42038331	-0.06167459
C	-1.94907227	1.54469231	-0.15608559
C	-1.15846227	0.34293831	-0.10292959
O	-1.81275527	-0.86928969	-0.01307559
C	0.23880373	0.22970931	-0.12273359
C	0.86646973	-1.05077069	-0.09097159
C	2.22500473	-1.19722869	-0.12079959
C	3.09599773	-0.07495269	-0.19362659
C	2.47357873	1.20678231	-0.19596159
C	1.11745373	1.35295331	-0.16479459
N	4.44410073	-0.22219869	-0.25890059
C	5.32320773	0.93699331	-0.14573559

C	5.54389673	1.37879731	1.29559741
C	5.01640173	-1.56622169	-0.10601459
C	6.51339673	-1.66383469	-0.33001959
O	-4.03336512	2.50648297	-0.10734214
O	-1.51865957	2.55386060	-0.25882822
H	-3.49259870	3.27248412	-0.18963051
H	-3.02567127	-3.09131069	0.16988241
H	-6.04684927	0.84134531	0.09151041
H	-6.99251227	-1.43398369	0.26447141
H	-5.48826327	-3.40380869	0.30334541
H	0.25213173	-1.93871569	-0.03863559
H	2.62609273	-2.19899469	-0.08564859
H	3.07444173	2.10383831	-0.21067359
H	0.69237673	2.34545631	-0.16877059
H	6.27420573	0.68519931	-0.60669959
H	4.91475873	1.75152931	-0.74115559
H	6.21091873	2.24293931	1.32249241
H	5.99946073	0.57685031	1.88020741
H	4.60165573	1.65760131	1.77130341
H	4.77475373	-1.94653169	0.89372341
H	4.52602473	-2.22349469	-0.82610559
H	6.79407473	-2.71435169	-0.23735659
H	7.08769573	-1.10306069	0.40844841
H	6.79847073	-1.33078169	-1.32942359

Computed SI coordinates of structure of DEAHF in [BMIm][PF_6] in N form (S_1 state)

C	-3.68426200	-2.26353500	0.36335100
C	-3.17953100	-0.97954700	0.16227100
C	-4.02586500	0.11690300	0.02240000
C	-5.41157900	-0.08569300	0.08755200

C	-5.91878900	-1.34767800	0.28557500
C	-5.04835900	-2.43734500	0.42308300
C	-3.43686700	1.42952200	-0.18834000
C	-1.99266400	1.46505200	-0.25031100
C	-1.23676100	0.34743200	-0.08791600
O	-1.83570300	-0.85291400	0.11825200
C	0.21914200	0.24214300	-0.13229500
C	0.82885100	-0.98202900	-0.41836500
C	2.20079900	-1.11839400	-0.45855900
C	3.05745800	-0.02161400	-0.22287600
C	2.43206700	1.20269300	0.09725200
C	1.06016300	1.32841400	0.13056400
N	4.42577700	-0.13968800	-0.31544200
C	5.27145500	0.90052300	0.25900000
C	5.36969100	0.84045500	1.77817600
C	4.98902500	-1.48690700	-0.43985600
C	6.48885000	-1.53291200	-0.66912000
O	-4.07906700	2.47502800	-0.33446000
O	-1.44541200	2.68070600	-0.48908300
H	-2.19826300	3.28799500	-0.58080700
H	-2.99807100	-3.09392000	0.46873600
H	-6.06571300	0.77009300	-0.02282500
H	-6.98876000	-1.50352600	0.33515500
H	-5.45110000	-3.43053600	0.57863500
H	0.21895700	-1.85477200	-0.61278900
H	2.60206600	-2.09586400	-0.67850800
H	3.02008900	2.07748600	0.33116200
H	0.63769800	2.29068000	0.38188500
H	6.26236500	0.80956400	-0.17916600
H	4.90442700	1.87356900	-0.06360900

H	6.01762200	1.63855700	2.14706800
H	5.78980600	-0.11471700	2.10178100
H	4.38869900	0.95578300	2.24383500
H	4.73968000	-2.08469200	0.44724300
H	4.51325700	-1.97170600	-1.29340400
H	6.77155300	-2.57104900	-0.85258100
H	7.05976200	-1.18492000	0.19277200
H	6.78000100	-0.94766500	-1.54368000

Computed SI coordinates of structure of DEAHF in [BMIm][PF_6] in T form (S_1 state)

C	-3.70980900	-2.26926900	0.16321300
C	-3.18380900	-0.99837300	0.05591200
C	-4.01783900	0.14338600	0.04360800
C	-5.41311800	-0.05392600	0.15422300
C	-5.93328400	-1.32653900	0.26418000
C	-5.08737700	-2.43522900	0.26995100
C	-3.39717100	1.39232000	-0.07018900
C	-1.97528000	1.51883600	-0.18705700
C	-1.18171300	0.31820500	-0.13171300
O	-1.83355400	-0.89701100	-0.05231900
C	0.21548800	0.20824100	-0.14212300
C	0.84508300	-1.07210200	-0.13093200
C	2.20371800	-1.21598700	-0.15408000
C	3.07357400	-0.09132000	-0.19544500
C	2.44948200	1.18925300	-0.17336800
C	1.09326800	1.33311800	-0.15027100
N	4.42264600	-0.23471700	-0.25456700
C	5.29767300	0.92015100	-0.07890800
C	5.48850500	1.30430700	1.38280800
C	4.99617200	-1.58090000	-0.12851600

C	6.49230000	-1.67194500	-0.35993700
O	-4.12120200	2.51908000	-0.11365900
O	-1.49866600	2.68009200	-0.32082000
H	-3.47684600	3.24317700	-0.21208500
H	-3.03920000	-3.11930100	0.16505700
H	-6.06420500	0.81061200	0.14874400
H	-7.00412000	-1.46468000	0.34761000
H	-5.49820000	-3.43344600	0.35520800
H	0.23200400	-1.96199900	-0.10200100
H	2.60581200	-2.21777000	-0.13663100
H	3.04898100	2.08722800	-0.16149100
H	0.66901200	2.32559700	-0.13382900
H	6.25788000	0.68765100	-0.53082400
H	4.90035200	1.75727700	-0.64988000
H	6.15542400	2.16563200	1.45760500
H	5.93107800	0.47951000	1.94526800
H	4.53667800	1.56559800	1.84933900
H	4.75751800	-1.98184200	0.86396800
H	4.50489200	-2.22355800	-0.86067300
H	6.77345500	-2.72464600	-0.29912200
H	7.06984900	-1.13319900	0.39218800
H	6.77331700	-1.30946300	-1.35023100

5. We employed the TZVP basis set along with PBEPBE functionals （with SMD）:

Computed SI coordinates of structure of DEAHF in [EMIm][NTf$_2$] in N form (S_0 state)

C	-3.73994700	-2.26475400	0.03757400
C	-3.20752900	-0.97725300	0.00893700
C	-4.03304000	0.14758300	0.03123000

C	-5.42179300	-0.03380700	0.07853600
C	-5.95628900	-1.30201900	0.10637100
C	-5.10950800	-2.41747100	0.08556800
C	-3.41465300	1.45819400	-0.00549800
C	-1.97352600	1.47415800	-0.03269100
C	-1.22932600	0.32822100	-0.06620300
O	-1.86254600	-0.87496100	-0.04469800
C	0.21480200	0.19597000	-0.10011700
C	0.82008800	-1.04459000	0.14631000
C	2.18769900	-1.20909900	0.11510400
C	3.05519600	-0.13460600	-0.18878500
C	2.44003700	1.11854800	-0.41107000
C	1.07202000	1.27312300	-0.37494900
N	4.41020700	-0.30530300	-0.27447500
C	5.28821400	0.85441800	-0.30088700
C	5.51217300	1.49071300	1.06226300
C	4.97495200	-1.60001600	0.10253600
C	6.43876800	-1.78109800	-0.24142900
O	-4.02491100	2.53727800	0.00613100
O	-1.42620100	2.70849500	-0.03178900
H	-2.20248200	3.30336000	-0.00834000
H	-3.07236600	-3.11843900	0.02023400
H	-6.05595500	0.84532800	0.09456600
H	-7.03062200	-1.44093700	0.14379000
H	-5.53223800	-3.41582900	0.10735000
H	0.20476800	-1.90440800	0.38146400
H	2.58393400	-2.18954700	0.33812800
H	3.03907300	1.99360700	-0.62198200
H	0.66054900	2.25512400	-0.56220900
H	6.24172900	0.54212800	-0.72372800

H	4.88645400	1.58621000	-1.00303500
H	6.18199400	2.35035600	0.97086600
H	5.96856300	0.78033600	1.75689700
H	4.57323400	1.83842600	1.50036300
H	4.82753300	-1.77993600	1.17719600
H	4.41326000	-2.37451200	-0.42505000
H	6.71654600	-2.81183900	-0.00788500
H	7.09651200	-1.12704100	0.33455500
H	6.62731600	-1.61906400	-1.30579700

Computed SI coordinates of structure of DEAHF in [EMIm][NTf_2] in T form (S_0 state)

C	-3.29029651	-2.07524292	0.03780013
C	-2.75766751	-0.78662892	0.00699413
C	-3.58355951	0.34038908	0.02358513
C	-4.97379351	0.15762308	0.06848613
C	-5.50848851	-1.11196492	0.09855913
C	-4.66131951	-2.22850292	0.08294713
C	-2.96321951	1.65155008	-0.01401487
C	-1.52107151	1.66795708	-0.03998587
C	-0.77264051	0.52174508	-0.06770987
O	-1.40924851	-0.68394092	-0.04408787
C	0.67213049	0.38855508	-0.09830387
C	1.27984649	-0.85052892	0.16103413
C	2.64848049	-1.01445192	0.13310413
C	3.51804949	0.05834908	-0.17961987
C	2.90037449	1.30966408	-0.41632487
C	1.53138849	1.46393508	-0.38310487
N	4.87550049	-0.11124992	-0.26136287
C	5.75701449	1.05042508	-0.29701987
C	5.98929949	1.69833008	1.06275113

C	5.44480649	-1.41322492	0.09922813
C	6.89970749	-1.60679192	-0.28603687
O	-3.57452851	2.73380508	-0.00450087
O	-0.97377551	2.90548808	-0.03981687
H	-2.66771449	3.11499292	-0.01156113
H	-2.62314951	-2.92900992	0.02458013
H	-5.60928651	1.03549708	0.08074313
H	-6.58284251	-1.25055992	0.13383913
H	-5.08390751	-3.22671792	0.10640413
H	0.66598849	-1.70855492	0.40476013
H	3.04396949	-1.99215692	0.36799413
H	3.49827849	2.18266408	-0.63723187
H	1.12047849	2.44342408	-0.58242387
H	6.70803449	0.73422008	-0.72154487
H	5.35377449	1.77847408	-1.00182287
H	6.65944949	2.55630508	0.95915713
H	6.44933949	0.99485208	1.76141513
H	5.05408149	2.05174608	1.50343913
H	5.32555049	-1.59426192	1.17671313
H	4.86330349	-2.18153792	-0.41449587
H	7.17287549	-2.64174492	-0.06598587
H	7.58024049	-0.96422192	0.27561113
H	7.06136549	-1.44084292	-1.35404787

Computed SI coordinates of structure of DEAHF in [EMIm][NTf_2] in N form (S_1 state)

C	-3.75075200	-2.25218000	0.16608000
C	-3.21473500	-0.98291600	0.07035400
C	-4.04135300	0.16298600	0.02003600
C	-5.44043200	-0.03093100	0.07353200
C	-5.97453200	-1.30307400	0.16914500

C	-5.13891500	-2.41650200	0.21603900
C	-3.43275600	1.44282200	-0.07514600
C	-1.97961400	1.45850700	-0.12341900
C	-1.20343100	0.31701600	-0.06464400
O	-1.85409900	-0.89845000	0.02555400
C	0.21646900	0.19659300	-0.09449700
C	0.82875800	-1.08419700	-0.05471700
C	2.18960900	-1.23776500	-0.08770500
C	3.05949600	-0.11717800	-0.16638700
C	2.44618400	1.16773600	-0.18615800
C	1.08740200	1.31613100	-0.15189000
N	4.40741700	-0.26784600	-0.21914100
C	5.29253700	0.88583100	-0.17802000
C	5.56893200	1.37431700	1.23702000
C	4.97859300	-1.61272800	-0.11002400
C	6.46915600	-1.71136300	-0.34335600
O	-4.00734900	2.57144000	-0.12982900
O	-1.49056400	2.69136300	-0.22135600
H	-2.33228000	3.22550100	-0.22859900
H	-3.08278900	-3.10547400	0.20175500
H	-6.07910800	0.84395500	0.03641600
H	-7.05089400	-1.43401200	0.20829500
H	-5.55647000	-3.41410200	0.29097000
H	0.20491000	-1.96590900	0.00420500
H	2.59047500	-2.24078000	-0.04707600
H	3.05137400	2.06268400	-0.21882700
H	0.67551500	2.31499600	-0.16791200
H	6.22508900	0.60652700	-0.66341000
H	4.86319800	1.68318400	-0.78405600
H	6.24440400	2.23280900	1.19967000

H	6.04251700	0.59314900	1.83616100
H	4.64804000	1.68259000	1.73713500
H	4.73586100	-2.01352700	0.88265600
H	4.47143600	-2.24943300	-0.83983400
H	6.74486100	-2.76483300	-0.25582300
H	7.05191600	-1.15679100	0.39437500
H	6.75242300	-1.37906900	-1.34452900

Computed SI coordinates of structure of DEAHF in [EMIm][NTf_2] in T form (S_1 state)

C	-3.72908600	-2.26220000	0.17345800
C	-3.19492700	-0.99191100	0.07462300
C	-4.03016000	0.15198300	0.01444100
C	-5.43346600	-0.04298900	0.05950200
C	-5.95738900	-1.31545600	0.15819700
C	-5.11354300	-2.42579300	0.21581000
C	-3.39787200	1.38979000	-0.08123600
C	-1.96286700	1.51461400	-0.12707400
C	-1.18901900	0.32085600	-0.06131700
O	-1.84571600	-0.88978100	0.03549000
C	0.21892300	0.19700100	-0.08475400
C	0.84209700	-1.07835400	-0.01798000
C	2.20527400	-1.22543500	-0.05021100
C	3.07232500	-0.10728600	-0.16201000
C	2.45068300	1.17157200	-0.19808800
C	1.09016300	1.31662600	-0.16608100
N	4.42339900	-0.25433800	-0.23594900
C	5.30166500	0.90203800	-0.15442300
C	5.55465000	1.36973800	1.27138400
C	4.99770700	-1.59344600	-0.10331300
C	6.47282000	-1.70022000	-0.42315400

O	-4.08118900	2.53201100	-0.14404700
O	-1.50252200	2.69013100	-0.22168300
H	-3.37985800	3.21742800	-0.21063500
H	-3.06009500	-3.11405900	0.21703300
H	-6.08192100	0.82397400	0.01455000
H	-7.03255900	-1.45367900	0.19150300
H	-5.52977000	-3.42384100	0.29339700
H	0.22685700	-1.96482100	0.06698500
H	2.60923400	-2.22604400	0.01809000
H	3.05186400	2.06895900	-0.24932800
H	0.65978600	2.30811400	-0.20106400
H	6.24431000	0.64048200	-0.63131100
H	4.88053600	1.70975800	-0.75361600
H	6.22361700	2.23456400	1.26353100
H	6.02500800	0.58214400	1.86544500
H	4.62452700	1.66191400	1.76460700
H	4.81642500	-1.96614900	0.91445300
H	4.45562300	-2.25681100	-0.78192700
H	6.75376200	-2.75337400	-0.34709500
H	7.10156300	-1.14248800	0.27346900
H	6.69484600	-1.37136100	-1.44117100

Computed SI coordinates of structure of DEAHF in [BMIm][PF_6] in N form (S_0 state)

C	-3.73661969	-2.26255960	0.03938252
C	-3.20420169	-0.97505860	0.01074552
C	-4.02971269	0.14977740	0.03303852
C	-5.41846569	-0.03161260	0.08034452
C	-5.95296169	-1.29982460	0.10817952
C	-5.10618069	-2.41527660	0.08737652
C	-3.41132569	1.46038840	-0.00368948

C	-1.97019869	1.47635240	-0.03088248
C	-1.22599869	0.33041540	-0.06439448
O	-1.85921869	-0.87276660	-0.04288948
C	0.21812931	0.19816440	-0.09830848
C	0.82341531	-1.04239560	0.14811852
C	2.19102631	-1.20690460	0.11691252
C	3.05852331	-0.13241160	-0.18697648
C	2.44336431	1.12074240	-0.40926148
C	1.07534731	1.27531740	-0.37314048
N	4.41353431	-0.30310860	-0.27266648
C	5.27490088	0.86438290	-0.30432189
C	5.49576569	1.50503664	1.05729053
C	4.95998004	-1.60250079	0.10113168
C	6.42280231	-1.79500709	-0.24085111
O	-4.02156052	2.53941664	0.00793811
O	-1.42289166	2.71066596	-0.02998063
H	-2.19179003	3.29993584	-0.00675347
H	-3.06903869	-3.11624460	0.02204252
H	-6.05262769	0.84752240	0.09637452
H	-7.02729469	-1.43874260	0.14559852
H	-5.52891069	-3.41363460	0.10915852
H	0.20809531	-1.90221360	0.38327252
H	2.58726131	-2.18735260	0.33993652
H	3.04240031	1.99580140	-0.62017348
H	0.66387631	2.25731840	-0.56040048
H	6.23028688	0.55917435	-0.72810928
H	4.86601099	1.59153200	-1.00717728
H	6.15833237	2.36999826	0.96323484
H	5.95909523	0.79973794	1.75250503
H	4.55468736	1.84581542	1.49624813

H	4.80936546	-1.78318442	1.17522030
H	4.39276951	-2.37136455	-0.42878311
H	6.69170381	-2.82843245	-0.00878552
H	7.08499165	-1.14745919	0.33738958
H	6.61438060	-1.63256686	-1.30461590

Computed SI coordinates of structure of DEAHF in [BMIm][PF_6] in T form (S_0 state)

C	-3.26646695	-2.06522594	0.03761460
C	-2.73383795	-0.77661194	0.00680860
C	-3.55972995	0.35040606	0.02339960
C	-4.94996395	0.16764006	0.06830060
C	-5.48465895	-1.10194794	0.09837360
C	-4.63748995	-2.21848594	0.08276160
C	-2.93938995	1.66156706	-0.01420040
C	-1.49724195	1.67797406	-0.04017140
C	-0.74881095	0.53176206	-0.06789540
O	-1.38541895	-0.67392394	-0.04427340
C	0.69596005	0.39857206	-0.09848940
C	1.30367605	-0.84051194	0.16084860
C	2.67231005	-1.00443494	0.13291860
C	3.54187905	0.06836606	-0.17980540
C	2.92420405	1.31968106	-0.41651040
C	1.55521805	1.47395206	-0.38329040
N	4.89933005	-0.10123294	-0.26154840
C	5.78084405	1.06044206	-0.29720540
C	6.01312905	1.70834706	1.06256560
C	5.46863605	-1.40320794	0.09904260
C	6.92353705	-1.59677494	-0.28622240
O	-3.55069895	2.74382206	-0.00468640
O	-0.94994595	2.91550506	-0.04000240

H	-2.69154405	3.10497594	-0.01137560
H	-2.59931995	-2.91899294	0.02439460
H	-5.58545695	1.04551406	0.08055760
H	-6.55901295	-1.24054294	0.13365360
H	-5.06007795	-3.21670094	0.10621860
H	0.68981805	-1.69853794	0.40457460
H	3.06779905	-1.98213994	0.36780860
H	3.52210805	2.19268106	-0.63741740
H	1.14430805	2.45344106	-0.58260940
H	6.73186405	0.74423706	-0.72173040
H	5.37760405	1.78849106	-1.00200840
H	6.68327905	2.56632206	0.95897160
H	6.47316905	1.00486906	1.76122960
H	5.07791105	2.06176306	1.50325360
H	5.34938005	-1.58424494	1.17652760
H	4.88713305	-2.17152094	-0.41468140
H	7.19670505	-2.63172794	-0.06617140
H	7.60407005	-0.95420494	0.27542560
H	7.08519505	-1.43082594	-1.35423340

Computed SI coordinates of structure of DEAHF in [BMIm][PF_6] in N form (S_1 state)

C	-3.74609384	-2.28255414	-0.23224062
C	-3.21170683	-1.00933883	-0.26160207
C	-4.04028127	0.13585498	-0.29187009
C	-5.43967678	-0.06306476	-0.28858310
C	-5.97213455	-1.33914820	-0.25938958
C	-5.13457111	-2.45173872	-0.23098969
C	-3.43340006	1.41977050	-0.31806680
C	-1.97952385	1.44050898	-0.32150998
C	-1.20163192	0.29927940	-0.28415424

O	-1.85063073	-0.92021129	-0.26167999
C	0.21891895	0.18321723	-0.27556761
C	0.83403766	-1.09680992	-0.26764614
C	2.19577359	-1.24599603	-0.26528942
C	3.06386091	-0.12128756	-0.27333563
C	2.44709999	1.16206888	-0.26114162
C	1.08741080	1.30604004	-0.26236067
N	4.41326148	-0.26682439	-0.29099918
C	5.27595887	0.86749302	-0.14303045
C	5.61254308	1.42825523	1.20727932
C	4.98540318	-1.61371798	-0.21759841
C	6.48267111	-1.69982913	-0.40923442
O	-4.00981020	2.54824766	-0.34571289
O	-1.49194534	2.67724589	-0.35593746
H	-2.33479262	3.20957644	-0.36772647
H	-3.07664399	-3.13513595	-0.20990074
H	-6.07988239	0.81121276	-0.31062244
H	-7.04874084	-1.47387009	-0.25814639
H	-5.55083744	-3.45242410	-0.20810513
H	0.21166802	-1.98151512	-0.26245737
H	2.59860519	-2.24895850	-0.25201041
H	3.04998086	2.05892360	-0.24015579
H	0.67283232	2.30386115	-0.25156133
H	6.20851087	0.58818902	-0.62842045
H	4.84661987	1.66484602	-0.74906645
H	6.28843457	2.28232956	1.11566219
H	6.10241321	0.66968319	1.82215479
H	4.70794481	1.75907746	1.72249572
H	4.71408179	-2.05388874	0.75070250
H	4.50272317	-2.22224181	-0.98697664

H	6.75914990	-2.75532967	-0.35489833
H	7.04095615	-1.17352215	0.36711932
H	6.79499679	-1.32768669	-1.38748262

Computed SI coordinates of structure of DEAHF in [BMIm][PF_6] in T form (S_1 state)

C	-3.73461912	-2.26762955	0.17398490
C	-3.20046012	-0.99734055	0.07514990
C	-4.03569312	0.14655345	0.01496790
C	-5.43899912	-0.04841855	0.06002890
C	-5.96292212	-1.32088555	0.15872390
C	-5.11907612	-2.43122255	0.21633690
C	-3.40340512	1.38436045	-0.08070910
C	-1.96840012	1.50918445	-0.12654710
C	-1.19455212	0.31542645	-0.06079010
O	-1.85124912	-0.89521055	0.03601690
C	0.21338988	0.19157145	-0.08422710
C	0.83656388	-1.08378355	-0.01745310
C	2.19974088	-1.23086455	-0.04968410
C	3.06679188	-0.11271555	-0.16148310
C	2.44514988	1.16614245	-0.19756110
C	1.08462988	1.31119645	-0.16555410
N	4.41786588	-0.25976755	-0.23542210
C	5.29613188	0.89660845	-0.15389610
C	5.54911688	1.36430845	1.27191090
C	4.99217388	-1.59887555	-0.10278610
C	6.46728688	-1.70564955	-0.42262710
O	-4.08672212	2.52658145	-0.14352010
O	-1.50584758	2.68408141	-0.22116909
H	-3.37653242	3.22347759	-0.21114891
H	-3.06562812	-3.11948855	0.21755990

H	-6.08745412	0.81854445	0.01507690
H	-7.03809212	-1.45910855	0.19202990
H	-5.53530312	-3.42927055	0.29392390
H	0.22132388	-1.97025055	0.06751190
H	2.60370088	-2.23147355	0.01861690
H	3.04633088	2.06352945	-0.24880110
H	0.65425288	2.30268445	-0.20053710
H	6.23877688	0.63505245	-0.63078410
H	4.87500288	1.70432845	-0.75308910
H	6.21808388	2.22913445	1.26405790
H	6.01947488	0.57671445	1.86597190
H	4.61899388	1.65648445	1.76513390
H	4.81089188	-1.97157855	0.91497990
H	4.45008988	-2.26224055	-0.78140010
H	6.74822888	-2.75880355	-0.34656810
H	7.09602988	-1.14791755	0.27399590
H	6.68931288	-1.37679055	-1.44064410

6. We employed the TZVP basis set along with B1B95 functionals （with SMD）:

Computed SI coordinates of structure of DEAHF in [EMIm][NTf_2] in N form (S_0 state)

C	-3.74057900	-2.25598100	-0.02840400
C	-3.20334900	-0.97335000	-0.02024400
C	-4.02103400	0.15096500	0.04154500
C	-5.40747800	-0.02414800	0.09289100
C	-5.94713300	-1.28706300	0.08497400
C	-5.10743600	-2.40307400	0.02401300
C	-3.39895900	1.45775300	0.04457700
C	-1.96074200	1.46906200	0.00682700

C	-1.22388400	0.32580500	-0.06866200
O	-1.85955900	-0.87426500	-0.08073000
C	0.21773200	0.18967900	-0.11275900
C	0.81744700	-1.04199300	0.16897400
C	2.18128500	-1.21332800	0.13293900
C	3.04741000	-0.15467200	-0.21227700
C	2.43749000	1.08893600	-0.47784200
C	1.07292700	1.25021900	-0.43435500
N	4.39915600	-0.33407600	-0.29225200
C	5.27500400	0.82051400	-0.40187800
C	5.47098300	1.56065700	0.90913500
C	4.95263500	-1.60416500	0.17295200
C	6.44126800	-1.76371200	-0.03458200
O	-4.00517600	2.53505000	0.10015000
O	-1.40644800	2.69849500	0.05209900
H	-2.17504200	3.29753800	0.10565200
H	-3.07900900	-3.10893600	-0.07670900
H	-6.03383300	0.85560300	0.14037200
H	-7.01864200	-1.42170600	0.12527400
H	-5.53377900	-3.39660400	0.01752300
H	0.20038200	-1.88736500	0.43549800
H	2.57534800	-2.18496700	0.38184600
H	3.03695600	1.95039600	-0.72442600
H	0.66513300	2.22283900	-0.65680100
H	6.23238800	0.48092700	-0.78400700
H	4.87955300	1.48867900	-1.16392000
H	6.12849700	2.41846200	0.76635600
H	5.92241900	0.90923800	1.65786300
H	4.52142600	1.92281000	1.30280000
H	4.71690100	-1.74538000	1.23389200

H	4.45041100	-2.40663900	-0.36784800
H	6.72200200	-2.76673800	0.28441800
H	7.02546200	-1.05666600	0.55235400
H	6.71727100	-1.65981700	-1.08345600

Computed SI coordinates of structure of DEAHF in [EMIm][NTf$_2$] in T form (S$_0$ state)

C	-3.26646695	-2.06522594	0.03761460
C	-2.73383795	-0.77661194	0.00680860
C	-3.55972995	0.35040606	0.02339960
C	-4.94996395	0.16764006	0.06830060
C	-5.48465895	-1.10194794	0.09837360
C	-4.63748995	-2.21848594	0.08276160
C	-2.93938995	1.66156706	-0.01420040
C	-1.49724195	1.67797406	-0.04017140
C	-0.74881095	0.53176206	-0.06789540
O	-1.38541895	-0.67392394	-0.04427340
C	0.69596005	0.39857206	-0.09848940
C	1.30367605	-0.84051194	0.16084860
C	2.67231005	-1.00443494	0.13291860
C	3.54187905	0.06836606	-0.17980540
C	2.92420405	1.31968106	-0.41651040
C	1.55521805	1.47395206	-0.38329040
N	4.89933005	-0.10123294	-0.26154840
C	5.78084405	1.06044206	-0.29720540
C	6.01312905	1.70834706	1.06256560
C	5.46863605	-1.40320794	0.09904260
C	6.92353705	-1.59677494	-0.28622240
O	-3.54383181	2.72785624	-0.00504809
O	-0.95551982	2.88336281	-0.04044620

H	-2.69149467	3.11645953	-0.01118857
H	-2.59931995	-2.91899294	0.02439460
H	-5.58545695	1.04551406	0.08055760
H	-6.55901295	-1.24054294	0.13365360
H	-5.06007795	-3.21670094	0.10621860
H	0.68981805	-1.69853794	0.40457460
H	3.06779905	-1.98213994	0.36780860
H	3.52210805	2.19268106	-0.63741740
H	1.14430805	2.45344106	-0.58260940
H	6.73186405	0.74423706	-0.72173040
H	5.37760405	1.78849106	-1.00200840
H	6.68327905	2.56632206	0.95897160
H	6.47316905	1.00486906	1.76122960
H	5.07791105	2.06176306	1.50325360
H	5.34938005	-1.58424494	1.17652760
H	4.88713305	-2.17152094	-0.41468140
H	7.19670505	-2.63172794	-0.06617140
H	7.60407005	-0.95420494	0.27542560
H	7.08519505	-1.43082594	-1.35423340

Computed SI coordinates of structure of DEAHF in [EMIm][NTf$_2$] in N form (S$_1$ state)

C	-3.75125200	-2.25636600	0.18096100
C	-3.21878800	-0.98487300	0.07711200
C	-4.04855400	0.16147600	0.01364400
C	-5.45085900	-0.03921300	0.05994100
C	-5.98005200	-1.31326300	0.16326200
C	-5.14093800	-2.42561600	0.22440600
C	-3.44205600	1.43981500	-0.09065500
C	-1.98066300	1.46160200	-0.12748700
C	-1.20738900	0.32528600	-0.06257500

O	-1.85427900	-0.89543800	0.03846700
C	0.22277600	0.20159800	-0.08737100
C	0.83289700	-1.07613500	-0.01691600
C	2.19645600	-1.23116000	-0.04755000
C	3.06621300	-0.11372000	-0.15403600
C	2.45283600	1.16922400	-0.20548400
C	1.09073600	1.31832600	-0.17454400
N	4.42061000	-0.26464500	-0.20783900
C	5.30580200	0.89195900	-0.13211400
C	5.55148800	1.36932300	1.29775900
C	4.99609600	-1.61291400	-0.11804100
C	6.47020300	-1.72392600	-0.45606800
O	-4.01903000	2.57076400	-0.15831900
O	-1.49420600	2.70087500	-0.22888000
H	-2.33697300	3.23235400	-0.24659500
H	-3.08055000	-3.10616300	0.22671800
H	-6.09361700	0.83125000	0.01197400
H	-7.05581600	-1.44755200	0.19706900
H	-5.55596500	-3.42316400	0.30522900
H	0.20863000	-1.95453400	0.06729200
H	2.59548900	-2.23205000	0.02568200
H	3.05572300	2.06307200	-0.27028000
H	0.67642900	2.31460800	-0.21983900
H	6.24812800	0.62033600	-0.60061600
H	4.89059700	1.69473100	-0.74008300
H	6.22206100	2.23092200	1.28058200
H	6.01672800	0.58703200	1.90017300
H	4.61982400	1.66793600	1.78140900
H	4.82118400	-2.00302200	0.89285800
H	4.43896600	-2.25265600	-0.80518000

H	6.73864800	-2.78087600	-0.40920900
H	7.11121600	-1.19406300	0.24934800
H	6.68543400	-1.37379200	-1.46727500

Computed SI coordinates of structure of DEAHF in [EMIm][NTf$_2$] in T form (S_1 state)

C	-3.73315300	-2.25143700	0.15735200
C	-3.20206800	-0.98251300	0.06947400
C	-4.02756400	0.15721300	0.02894000
C	-5.42109300	-0.03764900	0.08430000
C	-5.95278500	-1.30832500	0.17247100
C	-5.11631100	-2.41754000	0.21010300
C	-3.42397100	1.44047300	-0.05644100
C	-1.98037000	1.46218000	-0.11749100
C	-1.19696400	0.32419900	-0.06686800
O	-1.84309600	-0.89263100	0.02147500
C	0.21733200	0.20540600	-0.10939600
C	0.82366400	-1.07769200	-0.09872400
C	2.17991000	-1.23734800	-0.13611800
C	3.05323100	-0.12145900	-0.18933900
C	2.44793500	1.16442800	-0.18841800
C	1.09353300	1.31963100	-0.14834700
N	4.39741000	-0.27764600	-0.23566100
C	5.28159100	0.87589900	-0.20998500
C	5.51670000	1.40574300	1.19484300
C	4.95903900	-1.62426400	-0.10914400
C	6.46199300	-1.71496300	-0.21441200
O	-4.01837300	2.56042400	-0.09777800
O	-1.47940000	2.69124200	-0.21653400
H	-2.29626000	3.24842400	-0.21633600
H	-3.06458700	-3.10038500	0.18515300

H	-6.05895600	0.83437800	0.05358100
H	-7.02550200	-1.44088300	0.21323900
H	-5.53075200	-3.41340900	0.27934700
H	0.19690100	-1.95450000	-0.06099400
H	2.57536500	-2.23998000	-0.12403100
H	3.05659400	2.05377300	-0.20260000
H	0.69128000	2.31860100	-0.14221000
H	6.22341800	0.58329000	-0.66029900
H	4.86678700	1.64905100	-0.85159400
H	6.18272400	2.26712000	1.15406400
H	5.97866100	0.64541100	1.82339600
H	4.58171200	1.71505200	1.66019400
H	4.63463000	-2.04004000	0.84906600
H	4.51467000	-2.24579100	-0.88791800
H	6.73666800	-2.76096500	-0.08609200
H	6.97093700	-1.14289200	0.55916300
H	6.82689700	-1.39691200	-1.18961100

Computed SI coordinates of structure of DEAHF in [BMIm][PF_6] in N form (S_0 state)

C	3.74382300	-2.26882700	-0.05619900
C	3.21188900	-0.98001500	-0.01667200
C	4.03922400	0.14611900	-0.02654000
C	5.42922000	-0.03763300	-0.07342600
C	5.96300900	-1.30715200	-0.11233200
C	5.11460500	-2.42306400	-0.10334300
C	3.42050300	1.45704800	0.01974300
C	1.97916500	1.47577300	0.04620100
C	1.22848800	0.33000700	0.06708200
O	1.86386500	-0.87578300	0.03517800
C	-0.21602500	0.19812400	0.09772800

C	-0.82298300	-1.04434500	-0.14603100
C	-2.19142700	-1.20755100	-0.11821900
C	-3.06107200	-0.13054900	0.17730000
C	-2.44413600	1.12330700	0.39890300
C	-1.07513200	1.27749100	0.36647300
N	-4.41957300	-0.29928000	0.25880900
C	-5.29963700	0.86328400	0.26925400
C	-5.51546000	1.49585900	-1.10209000
C	-4.98594600	-1.60765700	-0.07935500
C	-6.44414200	-1.79617500	0.30028900
O	4.03388800	2.53988600	0.01762100
O	1.43500900	2.71582700	0.05602100
H	2.21713600	3.30465000	0.03972900
H	3.07541100	-3.12095800	-0.04807000
H	6.06346000	0.84066500	-0.08036000
H	7.03675500	-1.44640500	-0.14951100
H	5.53631500	-3.42097600	-0.13376800
H	-0.20707500	-1.90422500	-0.37584000
H	-2.58630900	-2.18788100	-0.34142200
H	-3.04177400	1.99947800	0.60606100
H	-0.66275000	2.25859600	0.55227500
H	-6.25546700	0.55517700	0.68843700
H	-4.90387200	1.59852700	0.97077900
H	-6.17891300	2.35993400	-1.01673200
H	-5.97385200	0.78590700	-1.79441900
H	-4.57262600	1.83381100	-1.53726100
H	-4.85962800	-1.81163500	-1.15202900
H	-4.40694100	-2.36454000	0.45374800
H	-6.71512800	-2.83490600	0.10053800
H	-7.12092300	-1.16688300	-0.27974100

H	-6.61270700	-1.60707500	1.36276200

Computed SI coordinates of structure of DEAHF in [BMIm][PF_6] in T form (S_0 state)

C	-3.26470710	-2.06440862	0.03756629
C	-2.73207810	-0.77579462	0.00676029
C	-3.55797010	0.35122338	0.02335129
C	-4.94820410	0.16845738	0.06825229
C	-5.48289910	-1.10113062	0.09832529
C	-4.63573010	-2.21766862	0.08271329
C	-2.93763010	1.66238438	-0.01424871
C	-1.49548210	1.67879138	-0.04021971
C	-0.74705110	0.53257938	-0.06794371
O	-1.38365910	-0.67310662	-0.04432171
C	0.69771990	0.39938938	-0.09853771
C	1.30543590	-0.83969462	0.16080029
C	2.67406990	-1.00361762	0.13287029
C	3.54363890	0.06918338	-0.17985371
C	2.92596390	1.32049838	-0.41655871
C	1.55697790	1.47476938	-0.38333871
N	4.90108990	-0.10041562	-0.26159671
C	5.78269185	1.06123301	-0.29715748
C	6.01496552	1.70908491	1.06264076
C	5.47043991	-1.40238551	0.09902960
C	6.92534091	-1.59595251	-0.28623540
O	-3.54207196	2.72867356	-0.00509640
O	-0.94987311	2.88366298	-0.04055994
H	-2.69722940	3.11614914	-0.01114546
H	-2.59756010	-2.91817562	0.02434629
H	-5.58369710	1.04633138	0.08050929
H	-6.55725310	-1.23972562	0.13360529

H	-5.05831810	-3.21588362	0.10617029
H	0.69157790	-1.69772062	0.40452629
H	3.06955890	-1.98132262	0.36776029
H	3.52386790	2.19349838	-0.63746571
H	1.14606790	2.45425838	-0.58265771
H	6.73371117	0.74500669	-0.72166813
H	5.37949711	1.78932001	-1.00194712
H	6.68514998	2.56703848	0.95909224
H	6.47496133	1.00556701	1.76129368
H	5.07974916	2.06252111	1.50331604
H	5.35127617	-1.58335878	1.17655145
H	4.88893691	-2.17069851	-0.41469440
H	7.19850891	-2.63090551	-0.06618440
H	7.60587391	-0.95338251	0.27541260
H	7.08699891	-1.43000351	-1.35424640

Computed SI coordinates of structure of DEAHF in [BMIm][PF_6] in N form (S_1 state)

C	-3.75125200	-2.25636600	0.18096100
C	-3.21878800	-0.98487300	0.07711200
C	-4.04855400	0.16147600	0.01364400
C	-5.45085900	-0.03921300	0.05994100
C	-5.98005200	-1.31326300	0.16326200
C	-5.14093800	-2.42561600	0.22440600
C	-3.44205600	1.43981500	-0.09065500
C	-1.98066300	1.46160200	-0.12748700
C	-1.20738900	0.32528600	-0.06257500
O	-1.85427900	-0.89543800	0.03846700
C	0.22277600	0.20159800	-0.08737100
C	0.83289700	-1.07613500	-0.01691600
C	2.19645600	-1.23116000	-0.04755000

C	3.06621300	-0.11372000	-0.15403600
C	2.45283600	1.16922400	-0.20548400
C	1.09073600	1.31832600	-0.17454400
N	4.42061000	-0.26464500	-0.20783900
C	5.30580200	0.89195900	-0.13211400
C	5.55148800	1.36932300	1.29775900
C	4.99609600	-1.61291400	-0.11804100
C	6.47020300	-1.72392600	-0.45606800
O	-4.01903000	2.57076400	-0.15831900
O	-1.49420600	2.70087500	-0.22888000
H	-2.33697300	3.23235400	-0.24659500
H	-3.08055000	-3.10616300	0.22671800
H	-6.09361700	0.83125000	0.01197400
H	-7.05581600	-1.44755200	0.19706900
H	-5.55596500	-3.42316400	0.30522900
H	0.20863000	-1.95453400	0.06729200
H	2.59548900	-2.23205000	0.02568200
H	3.05572300	2.06307200	-0.27028000
H	0.67642900	2.31460800	-0.21983900
H	6.24812800	0.62033600	-0.60061600
H	4.89059700	1.69473100	-0.74008300
H	6.22206100	2.23092200	1.28058200
H	6.01672800	0.58703200	1.90017300
H	4.61982400	1.66793600	1.78140900
H	4.82118400	-2.00302200	0.89285800
H	4.43896600	-2.25265600	-0.80518000
H	6.73864800	-2.78087600	-0.40920900
H	7.11121600	-1.19406300	0.24934800
H	6.68543400	-1.37379200	-1.46727500

Computed SI coordinates of structure of DEAHF in [BMIm][PF_6] in T form (S_1 state)

C	-3.73806561	-2.24808610	0.15735319
C	-3.20698061	-0.97916210	0.06947519
C	-4.03247661	0.16056390	0.02894119
C	-5.42600561	-0.03429810	0.08430119
C	-5.95769761	-1.30497410	0.17247219
C	-5.12122361	-2.41418910	0.21010419
C	-3.42888361	1.44382390	-0.05643981
C	-1.98528261	1.46553090	-0.11748981
C	-1.20187661	0.32754990	-0.06686681
O	-1.84800861	-0.88928010	0.02147619
C	0.21241939	0.20875690	-0.10939481
C	0.81875139	-1.07434110	-0.09872281
C	2.17499739	-1.23399710	-0.13611681
C	3.04831839	-0.11810810	-0.18933781
C	2.44302239	1.16777890	-0.18841681
C	1.08862039	1.32298190	-0.14834581
N	4.39249739	-0.27429510	-0.23565981
C	5.27667839	0.87924990	-0.20998381
C	5.51178739	1.40909390	1.19484419
C	4.95412639	-1.62091310	-0.10914281
C	6.45708039	-1.71161210	-0.21441081
O	-4.02328561	2.56377490	-0.09777681
O	-1.48431261	2.69459290	-0.21653281
H	-2.29134739	3.24507310	-0.21633719
H	-3.06949961	-3.09703410	0.18515419
H	-6.06386861	0.83772890	0.05358219
H	-7.03041461	-1.43753210	0.21324019
H	-5.53566461	-3.41005810	0.27934819
H	0.19198839	-1.95114910	-0.06099281

H	2.57045239	-2.23662910	-0.12402981
H	3.05168139	2.05712390	-0.20259881
H	0.68636739	2.32195190	-0.14220881
H	6.21850539	0.58664090	-0.66029781
H	4.86187439	1.65240190	-0.85159281
H	6.17781139	2.27047090	1.15406519
H	5.97374839	0.64876190	1.82339719
H	4.57679939	1.71840290	1.66019519
H	4.62971739	-2.03668910	0.84906719
H	4.50975739	-2.24244010	-0.88791681
H	6.73175539	-2.75761410	-0.08609081
H	6.96602439	-1.13954110	0.55916419
H	6.82198439	-1.39356110	-1.18960981

7. We employed the TZVP basis set along with B3PW91 functionals（without SMD）:

Computed SI coordinates of structure of DEAHF in gas in N form (S_0 state)

C	-3.73605000	-2.27058600	0.13833600
C	-3.20474300	-0.98326200	0.05361500
C	-4.03707600	0.13781700	0.02701900
C	-5.42532100	-0.04531100	0.08316100
C	-5.95685200	-1.31331400	0.16712200
C	-5.10597100	-2.42598700	0.19428500
C	-3.42535900	1.45113500	-0.06822000
C	-1.97765500	1.47261900	-0.10341500
C	-1.22530900	0.33159900	-0.08047500
O	-1.85790400	-0.87855900	-0.00103900

C	0.22148400	0.20216400	-0.11504900
C	0.83026000	-1.05014100	0.04342800
C	2.20089200	-1.20575000	0.01412700
C	3.06456100	-0.10940300	-0.19275100
C	2.44667700	1.15152300	-0.32847100
C	1.07649400	1.30050000	-0.29765600
N	4.43430800	-0.26470700	-0.27594900
C	5.29963500	0.89415300	-0.11269300
C	5.44938100	1.37161000	1.32939200
C	4.99774400	-1.59089500	-0.04159800
C	6.45577500	-1.74872100	-0.43986300
O	-4.03640300	2.52608300	-0.10771500
O	-1.45277000	2.71422400	-0.17407900
H	-2.24914500	3.28686500	-0.17979900
H	-3.06486700	-3.12040500	0.15713800
H	-6.05336100	0.83742700	0.05967200
H	-7.03039000	-1.45345400	0.21158300
H	-5.52517400	-3.42366800	0.25983300
H	0.21448800	-1.92508700	0.20440100
H	2.59764200	-2.19868100	0.16864800
H	3.04235400	2.04256100	-0.46798000
H	0.65895300	2.28988100	-0.41335700
H	6.27676300	0.64409400	-0.52364500
H	4.92629000	1.70243100	-0.74302800
H	6.09993300	2.24892000	1.37483900
H	5.89042000	0.59454300	1.95854000
H	4.48255700	1.64288700	1.75778800
H	4.87354200	-1.88947700	1.01093000
H	4.41807500	-2.30057800	-0.63598300
H	6.72859700	-2.80196100	-0.34455100

H	7.13695900	-1.18098400	0.19638300
H	6.62141900	-1.45484900	-1.47885300

Computed SI coordinates of structure of DEAHF in gas in T form (S_0 state)

C	-3.26470710	-2.06440862	0.03756629
C	-2.73207810	-0.77579462	0.00676029
C	-3.55797010	0.35122338	0.02335129
C	-4.94820410	0.16845738	0.06825229
C	-5.48289910	-1.10113062	0.09832529
C	-4.63573010	-2.21766862	0.08271329
C	-2.93763010	1.66238438	-0.01424871
C	-1.49548210	1.67879138	-0.04021971
C	-0.74705110	0.53257938	-0.06794371
O	-1.38365910	-0.67310662	-0.04432171
C	0.69771990	0.39938938	-0.09853771
C	1.30543590	-0.83969462	0.16080029
C	2.67406990	-1.00361762	0.13287029
C	3.54363890	0.06918338	-0.17985371
C	2.92596390	1.32049838	-0.41655871
C	1.55697790	1.47476938	-0.38333871
N	4.90108990	-0.10041562	-0.26159671
C	5.78269185	1.06123301	-0.29715748
C	6.01496552	1.70908491	1.06264076
C	5.47043991	-1.40238551	0.09902960
C	6.92534091	-1.59595251	-0.28623540
O	-3.53335046	2.71018994	-0.00552644
O	-0.95541792	2.84529096	-0.04110602
H	-2.69931442	3.13007059	-0.01088729
H	-2.59756010	-2.91817562	0.02434629
H	-5.58369710	1.04633138	0.08050929

H	-6.55725310	-1.23972562	0.13360529
H	-5.05831810	-3.21588362	0.10617029
H	0.69157790	-1.69772062	0.40452629
H	3.06955890	-1.98132262	0.36776029
H	3.52386790	2.19349838	-0.63746571
H	1.14606790	2.45425838	-0.58265771
H	6.73371117	0.74500669	-0.72166813
H	5.37949711	1.78932001	-1.00194712
H	6.68514998	2.56703848	0.95909224
H	6.47496133	1.00556701	1.76129368
H	5.07974916	2.06252111	1.50331604
H	5.35127617	-1.58335878	1.17655145
H	4.88893691	-2.17069851	-0.41469440
H	7.19850891	-2.63090551	-0.06618440
H	7.60587391	-0.95338251	0.27541260
H	7.08699891	-1.43000351	-1.35424640

Computed SI coordinates of structure of DEAHF in gas in N form (S_1 state)

C	-3.74951600	-2.25370600	0.19292100
C	-3.22238600	-0.98387200	0.07865600
C	-4.05545200	0.16360600	0.01585000
C	-5.46486500	-0.03930300	0.07395800
C	-5.98314500	-1.31176600	0.18724300
C	-5.14297100	-2.42546600	0.24770400
C	-3.45584000	1.43398000	-0.09838500
C	-1.95951600	1.45532000	-0.14172900
C	-1.21185400	0.32354000	-0.07844700
O	-1.84997400	-0.89636200	0.02815500
C	0.23202400	0.19727700	-0.10661600
C	0.83917200	-1.07260500	-0.02934400

C	2.20736800	-1.22436500	-0.05754600
C	3.06857400	-0.10832700	-0.16915500
C	2.45443600	1.16816800	-0.23151700
C	1.08830200	1.31341700	-0.20318500
N	4.43594500	-0.25400400	-0.22342200
C	5.30875400	0.90014700	-0.06539500
C	5.49389400	1.33170600	1.38819800
C	5.01013000	-1.59719100	-0.13684300
C	6.48001600	-1.70745800	-0.49973300
O	-3.99085800	2.56626200	-0.16888600
O	-1.50024000	2.69892300	-0.24639400
H	-2.37121700	3.20282800	-0.26043400
H	-3.07604300	-3.10095600	0.23740000
H	-6.10429200	0.83284000	0.02613100
H	-7.05840700	-1.44944000	0.22986000
H	-5.55696000	-3.42239800	0.33633100
H	0.20916500	-1.94639900	0.06055200
H	2.60845000	-2.22409000	0.02547100
H	3.05412300	2.06389900	-0.30731700
H	0.66196000	2.30506900	-0.25807300
H	6.27233200	0.65474000	-0.50673800
H	4.91383300	1.72355800	-0.66051700
H	6.15050700	2.20346700	1.43561800
H	5.94685700	0.53530600	1.98249200
H	4.53907800	1.59627300	1.84554800
H	4.85207000	-1.99756000	0.87469900
H	4.44446700	-2.23685000	-0.81757400
H	6.75321500	-2.76428500	-0.48116700
H	7.13499200	-1.19303200	0.20502800
H	6.68007300	-1.33411900	-1.50617000

Computed SI coordinates of structure of DEAHF in gas in T form (S_1 state)

C	-3.72521500	-2.26769500	0.19379100
C	-3.19709200	-0.99274800	0.07728600
C	-4.03696700	0.14325900	0.01703900
C	-5.43612600	-0.05097500	0.07273600
C	-5.95707400	-1.32726300	0.18724000
C	-5.11058100	-2.43263800	0.24731900
C	-3.40006100	1.38687200	-0.10220300
C	-1.96878000	1.50332700	-0.14035100
C	-1.18796000	0.32270900	-0.07855800
O	-1.84343400	-0.88520800	0.02552400
C	0.23063600	0.19969400	-0.11041100
C	0.85388100	-1.06559400	-0.00930300
C	2.22333200	-1.20739900	-0.03845700
C	3.08128900	-0.09472200	-0.18237000
C	2.45564900	1.17381500	-0.26320000
C	1.09044700	1.31677400	-0.23465800
N	4.45219200	-0.23470200	-0.26039900
C	5.31228400	0.91733000	-0.03657100
C	5.45743000	1.31334000	1.43123200
C	5.02581400	-1.57037700	-0.12858200
C	6.48495400	-1.69051800	-0.53522400
O	-4.08395100	2.52535800	-0.16958200
O	-1.50888800	2.69268900	-0.23306200
H	-3.37713700	3.20756800	-0.23718300
H	-3.05172700	-3.11462800	0.23837300
H	-6.08168800	0.81666900	0.02493700
H	-7.03101100	-1.46775100	0.22956700
H	-5.52390800	-3.43043700	0.33703400

H	0.24054900	-1.94928400	0.10635100
H	2.62834500	-2.20366300	0.06839400
H	3.04971300	2.07201600	-0.35873000
H	0.65356800	2.30327400	-0.30656600
H	6.29062800	0.69375400	-0.45914300
H	4.93389400	1.75799300	-0.61944800
H	6.10471200	2.18923500	1.52489000
H	5.90040000	0.50407100	2.01669900
H	4.48816500	1.55696500	1.87034900
H	4.90252500	-1.94461600	0.89973400
H	4.44735700	-2.23684700	-0.77224900
H	6.76193500	-2.74676500	-0.51474400
H	7.16249600	-1.16705900	0.14152400
H	6.65086500	-1.32344300	-1.55056000

Table S1. The parameters of ionic liquids $[EMIm][NTf_2]$ and $[BMIm][PF_6]$.

	$[EMIm][NTf_2]$	$[BMIm][PF_6]$
eps	12.25	11.40
epsinf	2.024	1.985
HBondAcidity	0.1002	0.266
HBondBasicity	0.2517	0.216
SurfaceTensionAtInterface	56.13	70.24
CarbonAromaticity	0.130	0.1765
ElectronegativeHalogenicity	0.261	0.3529
